# Brassinosteroids regulate root meristem development by mediating BIN2-UPB1 module in *Arabidopsis*

**DOI:** 10.1371/journal.pgen.1008883

**Published:** 2020-07-01

**Authors:** Taotao Li, Wei Lei, Ruiyuan He, Xiaoya Tang, Jifu Han, Lijuan Zou, Yanhai Yin, Honghui Lin, Dawei Zhang

**Affiliations:** 1 Key Laboratory of Bio-Resource and Eco-Environment of Ministry of Education, State Key Laboratory of Hydraulics and Mountain River Engineering, College of Life Sciences, Sichuan University, Chengdu, Sichuan, P. R. China; 2 Ecological Security and Protection Key Laboratory of Sichuan Province, Mianyang Normal University, Mianyang, Sichuan, P. R. China; 3 Department of Genetics, Development, and Cell Biology, Iowa State University, Ames, Iowa, United States of America; National University of Singapore and Temasek Life Sciences Laboratory, SINGAPORE

## Abstract

Plant steroid hormones brassinosteroids (BRs) regulate plant growth and development at many levels. While negative regulatory factors that inhibit development and are counteracted by BRs exist in the root meristem, these factors have not been characterized. The functions of UPB1 transcription factor in BR-regulated root growth have not been established, although its role in regulating root are well documented. Here, we found that BIN2 interacts with and phosphorylates the UPB1 transcription factor consequently promoting UPB1 stability and transcriptional activity. Genetic analysis revealed that UPB1 deficiency could partially recover the short-root phenotype of BR-deficient mutants. Expression of a mutated UPB1^S37AS41A^ protein lacking a conserved BIN2 phosphorylation sites can rescue shorter root phenotype of *bin2-1* mutant. In addition, *UPB1* was repressed by BES1 at the transcriptional level. The paclobutrazol-resistant protein family (PRE2/3) interacts with UPB1 and inhibits its transcriptional activity to promote root meristem development, and BIN2-mediated phosphorylation of UPB1 suppresses its interaction with PRE2/3, and subsequently impairing root meristem development. Taken together, our data elucidate a molecular mechanism by which BR promotes root growth via inhibiting BIN2-UPB1 module.

## Author summary

Various physiological and genetic researches have provided ample evidence in support of the role of plant hormones in root development. Brasinosteroids (BR) play important roles in controlling root growth and development, but the mechanism of negatively regulating factors in this process is less well studied. Here, we found BIN2 kinase, a negative component in BR signaling, interacted with and phosphorylated UPB1 to stabilize and promote its binding ability. The *upb1-1* mutant was hypersensitive phenotype, while *UPB1*-overexpression lines showed opposite effects on BR regulated root meristem development, and defect of *UPB1* partially suppressed the short-root phenotype in BR-deficient mutants. Moreover, the paclobutrazol-resistant protein family (PRE2/3) interacted with UPB1 and inhibited its transcriptional activity, and this interaction was also inhibited by BIN2 phosphorylation, thus impairing root meristem development. Our findings provide significant insights into BR signaling through BIN2-UPB1 in regulating root meristem.

## Introduction

Plant survival largely relies on an appropriate root system due to its mechanical supporting role and its function in water and nutrient acquisition. Phytohormones, including cytokinin, auxin, gibberellins, and abscisic acid, play an important role in regulating root development [1]. Brassinosteroids (BRs), a type of steroid hormone, have been shown to participate in different developmental processes, including cell expansion, leaf senescence, vascular development, photomorphogenesis, and stress responses [2–4]. BR signaling pathway has been elucidated. BRASSINOSTEROID-INSENSITIVE 1 (BRI1) is a receptor kinase that primarily perceives BR signal. BRI1 interacts with co-receptor BRI1-ASSOCIATED RECEPTOR KINASE (BAK1) and its homolog SOMATIC EMBRYOGENESISRECEPTOR KINASEs (SERKs) to from a more active BR receptor complex [5, 6]. The activated BRI1 phosphorylates BR-SIGNALING KINASE 1 (BSK1) and CONSTITUTIVE DEFFERENTIAL GROWTH 1 (CDG1), and these kinases positively regulate BRI1-SUPPRESSOR 1 (BSU1) by phosphorylation. Phosphorylated BSU1 can dephosphorylate and inactivate BRASSINOSTEROID INSENSITIVE 2 (BIN2), a glycogen synthase kinase 3-like kinase to regulate transcription factors BRI1-EMS-SUPRESSOR 1 (BES1) and BRASSINAZOLE RESISTANT 1 (BZR1), which promotes unphosphorylated BES1 and BZR1 accumulation in the cell nucleus, repressing or promoting target gene expression [7–9].

BRs have been demonstrated to regulate root growth. BR-related mutants display short roots, short hypocotyls, dwarfism, dark-green leaves, and reduced fertility, suggesting that BR were positively affect root growth [10]. BRs affect root elongation in a concentration-dependent manner [11]. BR can promote root meristem elongation by activating the cell cycle and cell differentiation progression [12]. In addition, transcriptomic analysis indicates that BR-induced genes were primarily detected in epidermal cells of basal meristem zone and repressed BR genes prevailed in the stele of the apical meristem zone [13]. Moreover, comparison between BR-responsive, BZR1-targeted, and developmental zone-specific transcriptomes indicates that BZR1 mostly activates its target genes expressed in the transition-elongation zone, but represses genes in the Quiescent central (QC) and surrounding stem cells [14]. Despite these impressive advances in root development research, the important molecular components for BR-mediated root meristem development are still unclear.

Members of the basic/helix-loop-helix (bHLH) family of transcription factors have been found in mammals, eukaryotes, and plants [15]. This family of transcription factors (TFs) has a well-known bHLH signature domain, which consists of approximately 60 amino acids with two functionally distinct regions. BZR1 and BES1 have atypical bHLH DNA-binding domains that can directly bind the SMALL AUXIN UP RNA (SAUR) family and paclobutrazol-resistant protein family (PRE) genes to promote cell elongation [16, 17]. bHLH093 and bHLH061 are also reported to be required for apical meristem function [18, 19]. UPBEAT1 (UPB1), as a bHLH subfamily 14 transcription factor, shows less homology to other members in the ATBS1-INTERACTING FACTOR (AIF) subfamily and modulates the balance between cell proliferation and differentiation by directly controlling the expression of peroxidases genes in root meristem [20]. However, its role in hormone-regulated plant growth remains to be explored.

We herein demonstrated that BIN2 enhanced the negative effect of BR signaling on root meristem development by directly interacting with and phosphorylating UPB1. Genetic experiments revealed that *UPB1* acted as a downstream component in BR signaling. In addition, we found that PRE2/3 could interact with UPB1 to influence root meristem formation. Our results thus revealed novel insights into the molecular mechanism underlying the link between BR signaling and root meristem development.

## Results

### BRs mediate root meristem development through BIN2 and BES1/BZR1

**Fig 1:**
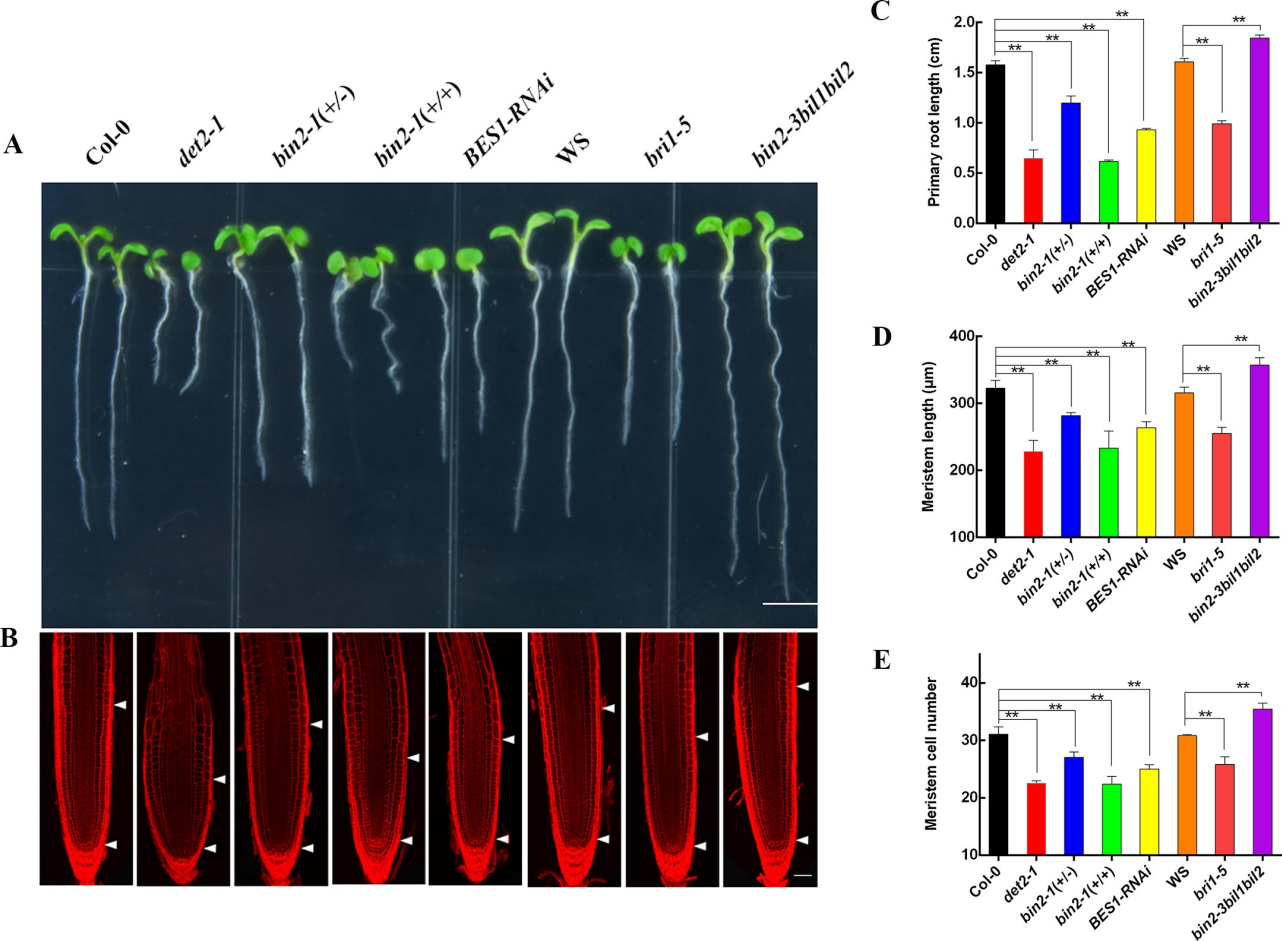
The root meristem phenotype is altered in the BR-related mutants. (A) Phenotypes of 5-day-old seedlings of Col-0, *det2-1*, *bin2-1*(+/-), *bin2-1*(+/+), *BES1-RNAi*, WS, *bri1-5*, and *bin2-3bil1bil2*. Bar = 0.5 cm. (B) Root meristem of Col-0, *det2-1*, *bin2-1*(+/-), *bin2-1*(+/+), *BES1-RNAi*, WS, *bri1-5*, and *bin2-3bil1bil2* in 5-day-old seedlings. White arrowheads (below) mark the position of the quiescent center (QC), and white arrowheads (above) mark the end of the meristem where cells start to elongate. Bar = 50 μm. Primary root length (C), meristem size (D), and meristem cell number (E) of the seedlings shown in (A). *bri1-5* and *bin2-3bil1bil2* are on the WS background, and other materials are on the Col-0 background. Date means ± SD (n≥20). Double asterisk represent highly significant differences (**, P<0.01; Student’s *t* test).

To evaluate the role of BRs on root meristem growth, we systematically examined the root meristem phenotypes in BR biosynthetic mutants, *det2-1*, and BR-responsive mutants, including the *bri1-5*, *bin2-1*(+/-) (*BIN2* gain-of-function), *bin2-1*(+/+), *bin2-3bil1bil2* (a triple knockout mutant of *BIN2* and its two closest homologs), and *BES1-RNAi* lines. The primary root lengths were shorter in *det2-1*, *bri1-5*, *bin2-1*(+/-), *bin2-1*(+/+), and *BES1-RNAi*, and significantly longer in *bin2-3bil1bil2* compared with those in their corresponding wild-type Col-0 or WS-2 plants; similar results were obtained with the meristem length and cell number (Fig 1A–1E). Combined with those of previous studies suggest that BR treatments increased root meristem size in lower concentrations, and BR dependent on the BZR1-mediated transcriptional responses in regulating root meristem [12, 14]. Thus, these results showed that BRs promote root meristem development dependent on BIN2 and/or its downstream components BZR1/BES1.

### BIN2 interacts with and phosphorylates UPB1

**Fig 2:**
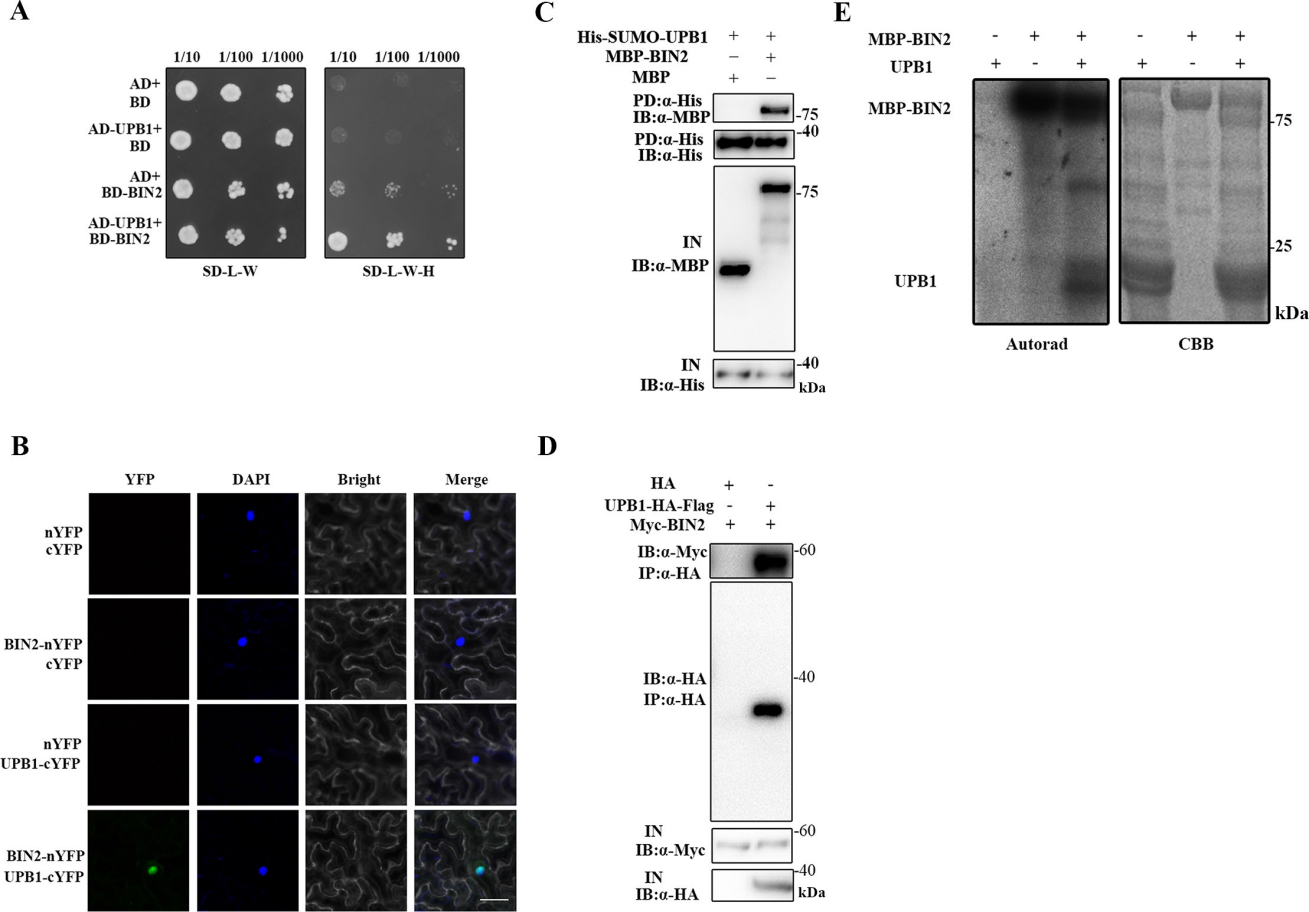
BIN2 interacts with and phosphorylates UPB1. (A) Y2H analyses the interaction between BIN2 and UPB1. Transformed yeast cells were grown on the SD-L-W or SD-L-W-H medium. (B) BiFC assays. The nYFP and cYFP, BIN2-nYFP and cYFP, nYFP and UPB1-cYFP, and BIN2-nYFP and UPB1-cYFP plasmids were co-transformed into *N*. *benthamiana* leaves cells. DAPI staining was used as a nuclear marker. Bar = 50 μm. (C) His pull-down assays of BIN2 and UPB1. His-SUMO-UPB1, MBP-BIN2, and MBP were purified from *E*. *coli*. Purified proteins were used for the pull-down assay. His-SUMO-UPB1 protein was detected with an anti-His antibody, MBP-BIN2 and MBP proteins were detected with anti-MBP antibodies. (D) Co-IP assays of BIN2 and UPB1. Protein extracts from 4-week-old *35S*::*BIN2-Myc/35S*::*UPB1-HA-Flag* and *35S*::*BIN2-Myc/35S*::*HA-Flag* seedlings, An anti-HA antibody was used for immunoprecipitation, and immunoblot analysis was performed with anti-HA and anti-Myc antibodies. (E) BIN2 phosphorylates UPB1 *in vitro*. The left blot shows autoradiography. The right blot shows the Coomassie brilliant blue (CBB) staining to indicate the loading of recombinant proteins.

To verify the molecular mechanism by which BIN2 regulates root meristem development, we used Y2H system to screen root meristem development-related components that interact with BIN2 and identified one clone encoding the bHLH protein UPB1. An independent Y2H experiment also confirmed that BIN2 could interact with UPB1 (Fig 2A). We further confirmed the result using a BiFC assay and found that the interaction occurred in the nucleus (Fig 2B). In addition, His pull-down assays indicated that BIN2 interacts with UPB1 *in vitro* (Fig 2C). Moreover. we generated *Arabidopsis* transgenic lines expressing *35S*::*BIN2-Myc* crossed with *35S*::*UPB1-HA-Flag* or *35S*::*HA-Flag* to obtain *35S*::*BIN2-Myc* on the *35S*::*UPB1-HA-Flag* and *35S*::*HA-Flag* backgrounds, respectively. Co-IP analysis revealed that BIN2 was associated with UPB1 *in planta* (Fig 2D). To explore whether UPB1 is a substrate of BIN2 kinase, we then performed *in vitro* kinase assays with ^32^P-γ-labeled ATP, and UPB1 could be phosphorylated by BIN2 (Fig 2E). However, we did not detected the interaction between BIL1/BIL2 and UPB1 in BiFC and Y2H assays, demonstrating that BIL1 and BIL2 could not interact with UPB1 (S1A and S1B Fig). Thus, these data demonstrated that BIN2 interacts with and phosphorylates the UPB1 protein.

### BR signaling mediates UPB1-regulated root meristem development

**Fig 3:**
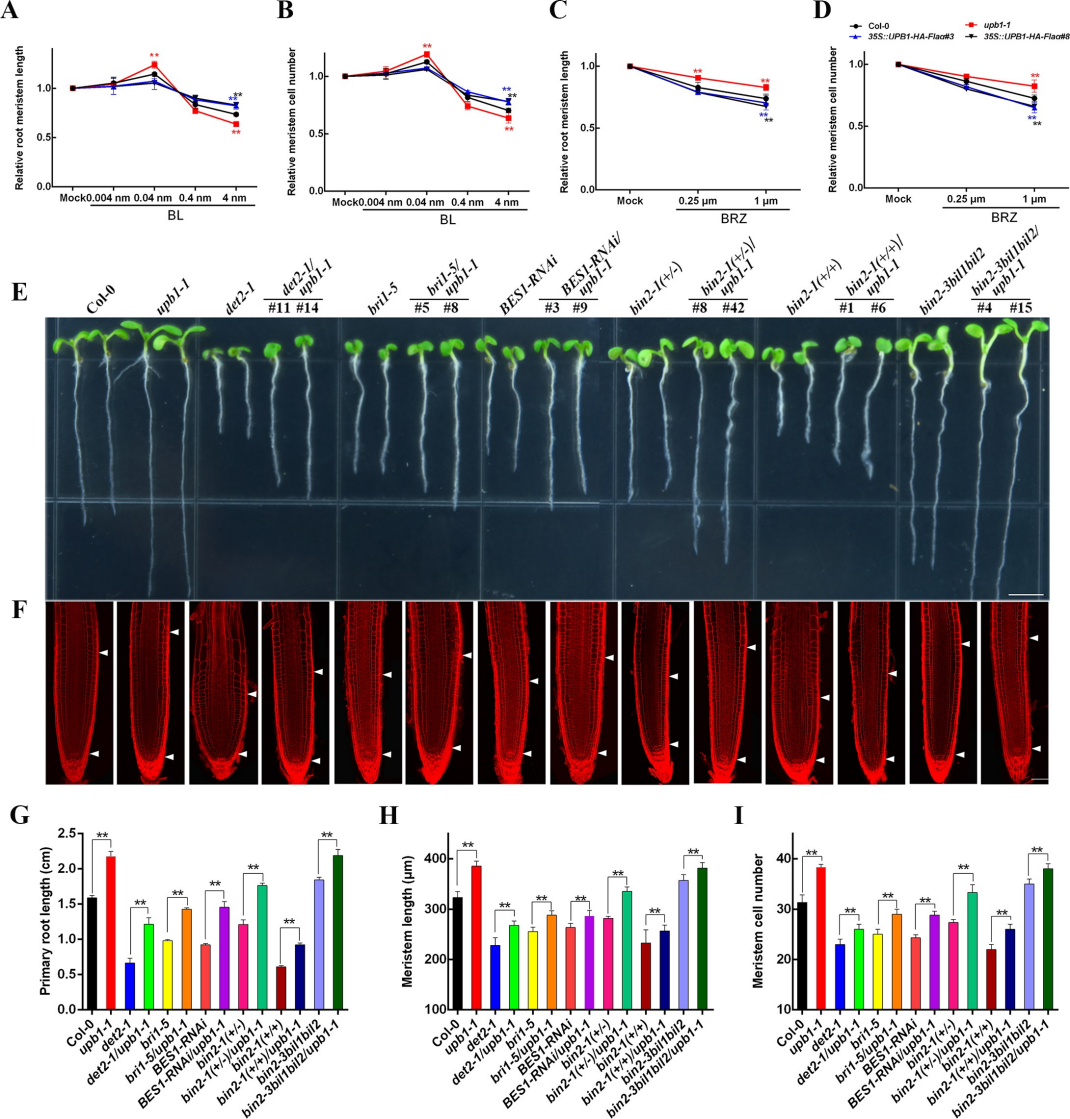
UPB1 as a negative regulator in the BR signaling pathway. (A, B) The root meristem size and cell number of 5-day-old Col-0, *upb1-1*, *35S*::*UPB1-HA-Flag#3*, and *35S*::*UPB1-HA-Flag#8* seedlings in the absence or presence of different concentrations of BL for another day. (C, D) The root meristem size and cell number of 5-day-old Col-0, *upb1-1*, *35S*::*UPB1-HA-Flag#3*, and *35S*::*UPB1-HA-Flag#8* seedlings in the absence or presence of different concentrations of BRZ for another day. (E) Phenotypes of 5-day-old seedlings of Col-0, *upb1-1*, *det2-1*, *det2-1/upb1-1*, *bri1-5*, *bri1-5/upb1-1*, *BES1-RNAi*, *BES1-RNAi/upb1-1*, *bin2-1*(+/-), *bin2-1*(+/-)*/upb1-1*, *bin2-1*(+/+), *bin2-1*(+/+)*/upb1-1*, *bin2-3bil1bil2*, and *bin2-3bil1bil2/upb1-1*. Bar = 0.5 cm. (F) Root meristem of Col-0, *upb1-1*, *det2-1*, *det2-1/upb1-1*, *bri1-5*, *bri1-5/upb1-1*, *BES1-RNAi*, *BES1-RNAi/upb1-1*, *bin2-1*(+/-), *bin2-1*(+/-)*/upb1-1*, *bin2-1*(+/+), *bin2-1*(+/+)*/upb1-1*, *bin2-3bil1bil2*, and *bin2-3bil1bil2/upb1-1* in 5-day-old seedlings. White arrowheads (below) mark the position of the quiescent center (QC), and white arrowheads (above) mark the end of the meristem where cells start to elongate. Bar = 50 μm. The primary root length (G), meristem size (H), and meristem cell number (I) of the seedlings shown in (E). Date means ± SD (n≥20). Double asterisk represent highly significant differences (**, P<0.01; Student’s *t* test).

To determine the biological function of *UPB1* in the BR signaling pathway, we analyzed the *pUPB1-YFP* transgenic plants, and fluorescent signals were observed in the leaf vein and root (S2A Fig). Moreover, we also obtained *35*::*UPB1-HA-Flag* transgenic plants on the *upb1-1* background, which displayed reduced rosettes, rounded leaves, dark green color, and reduced fertility, especially in those with short roots (S2B–S2H Fig). Expression analysis revealed that *UPB1* expression was gradually reduced in root meristem by exogenous application BL (S3A Fig). In addition, our data showed that the relative transcript levels of *UPB1* in the BR-biosynthetic defection mutant, *det2-1*, and BR-responsive insensitive mutants, including *bri1-5* and *BES1-RNAi*, were significantly enhanced when compared with the wild-type (S3B Fig). Interestingly, the expression level of *UPB1* in *bin2-1*(+/-) and *bin2-1*(+/+) was not significantly changed compared with that in the control. Moreover, previous research revealed the *phyB activation-tagged suppressor 1* (*BAS1*) is a well-known indicator representing the negative feedback regulation signal in BR metabolic pathway [21]. The expression of *BAS1* was no significantly change in Col-0, *upb1-1*, *35S*::*UPB1-HA-Flag#3*, and *35S*::*UPB1-HA-Flag#8* seedlings (S3C Fig), suggesting that *UPB1* was not a component in feedback regulation of BR homeostasis. To confirm that the *35S*::*UPB1-HA-Flag* phenotype is related to the BR pathway, we assessed Col-0, *upb1-1*, and *35S*::*UPB1-HA-Flag* seedlings treated with different concentrations of the brassinolide (BL) and BR biosynthesis inhibitor brassinazole (BRZ), which reduces endogenous BR levels [22]. It shows BL promoted root growth only at very low concentrations (0.04 nM), BL concentrations higher than 0.04 nM inhibited root growth and meristem size. The *upb1-1* plants showed higher sensitive and *35S*::*UPB1-HA-Flag* plants were less sensitive to exogenous different concentrations of BL in regulating root meristem length and cell number than wild type. (Fig 3A). Conversely, *35S*::*UPB1-HA-Flag* seedlings had short meristem and were more sensitive to BRZ than wild-type seedlings. The *upb1-1* showed a reduced response to BRZ compared with that of the wild-type seedlings (Fig 3C). Likewise, root meristem cell number showed similar response to BRZ (Fig 3B and 3D). These evidence reveals that UPB1 plays a negative role in the BR signaling pathway in regulating root meristem development.

We further examined the genetic interactions between *UPB1* and the *DET2*, *BRI1*, *BES1*, and *BIN2* genes. We crossed the *upb1-1* mutant with the *det2-1*, *bri1-5*, *BES1-RNAi*, *bin2-1*(+/-), and *bin2-3bil1bil2* mutants, resulting in *det2-1/upb1-1*, *bri1-5/upb1-1*, *BES1-RNAi/upb1-1*, *bin2-1*(+/-)*/upb1-1*, *bin2-1*(+/+)*/upb1-1*, and *bin2-3bil1bil2/upb1-1* multiple mutants (Fig 3E). The primary root length, meristem size, and cell number of *det2-1*, *bri1-5*, *BES1-RNAi*, *bin2-1*(+/-), and *bin2-1*(+/+) were partially rescued by *upb1-1* (Fig 3F–3I). Moreover, the long meristem phenotype of *bin2-3bil1bil2* was partially increased in *bin2-3bil1bil2/upb1-1*. This genetic evidence clearly demonstrated that UPB1 is functionally downstream of BR signaling.

### BIN2 enhances regulation in root meristem development through UPB1

**Fig 4:**
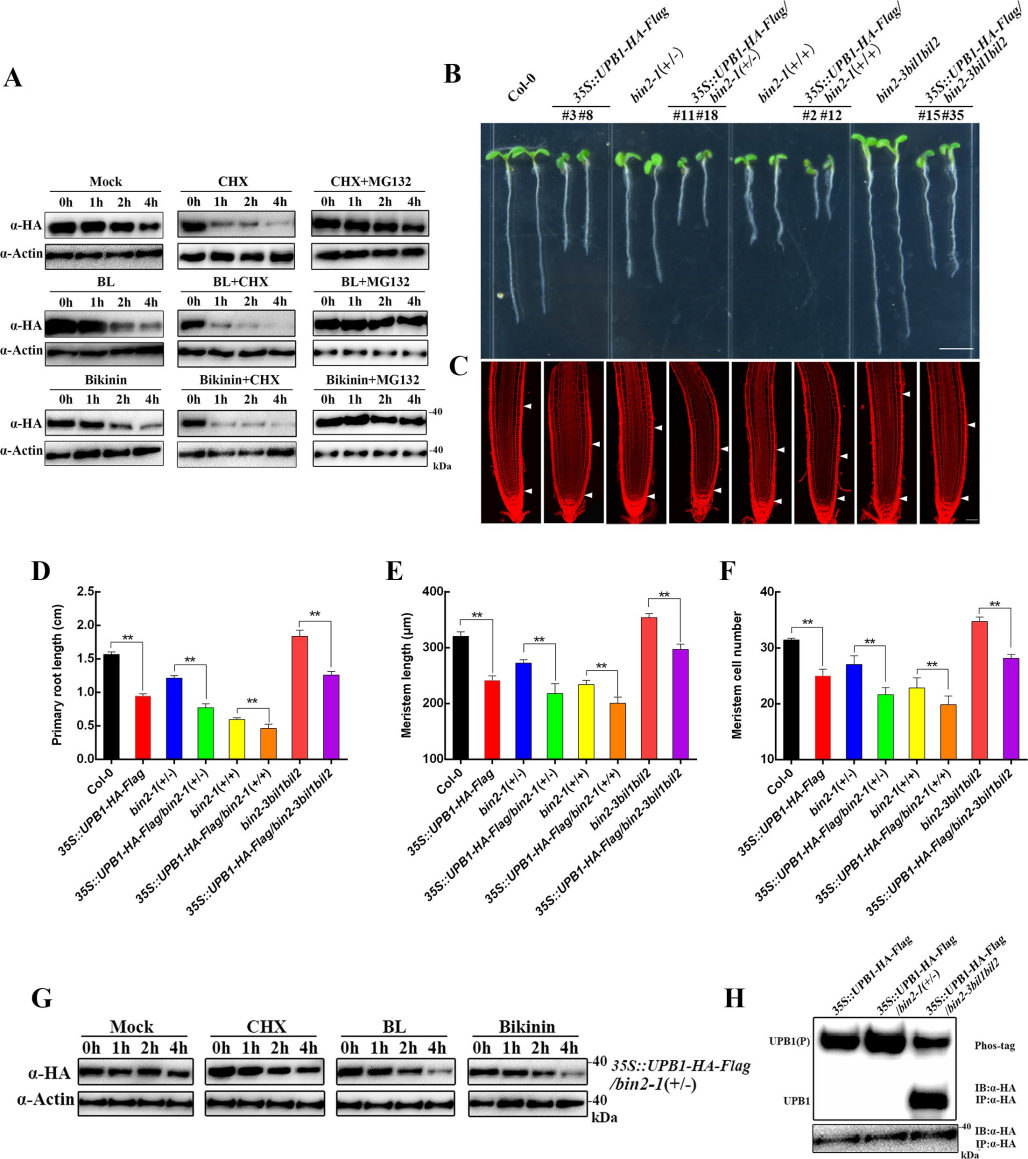
BIN2 regulates the stability of UPB1. (A) *In vivo* UPB1 degradation assays. Ten-day-old *35S*::*UPB1-HA-Flag* transgenic seedlings was treated without or with 1 μM BL or 50 μM bikinin and then incubated in the same medium supplemented with 100 μM CHX or 50 μM MG132. Samples were collected at indicated time points. UPB1 was detected with an anti-HA antibody. Actin was used as a control. (B) Phenotypes of 5-day-old seedlings of Col-0, *35S*::*UPB1-HA-Flag*, *bin2-1*(+/-), *35S*::*UPB1-HA-Flag/bin2-1*(+/-), *bin2-1*(+/+), *35S*::*UPB1-HA-Flag/bin2-1*(+/+), *bin2-3bil1bil2*, and *35S*::*UPB1-HA-Flag/bin2-3bil1bil2*. Bar = 0.5 cm. (C) Root meristem of Col-0, *35S*::*UPB1-HA-Flag*, *bin2-1*(+/-), *35S*::*UPB1-HA-Flag/bin2-1*(+/-), *bin2-1*(+/+), *35S*::*UPB1-HA-Flag/bin2-1*(+/+), *bin2-3bil1bil2*, and *35S*::*UPB1-HA-Flag/bin2-3bil1bil2* in 5-day-old seedlings. White arrowheads (below) mark the position of the quiescent center (QC), and white arrowheads (above) mark the end of the meristem where cells start to elongate. Bar = 50 μm. The primary root length (D), meristem size (E), and meristem cell number (F) in the seedlings shown in (B). Date means ± SD (n≥20). Double asterisk represent highly significant differences (**, P<0.01; Student’s *t* test). (G) *In vivo* UPB1 degradation assays. Ten-day-old *35S*::*UPB1-HA-Flag/bin2-1*(+/-) transgenic seedlings was treated without or with 100 μM CHX, 1 μM BL, and 50 μM bikinin. Samples were collected at the indicated time points. UPB1 was detected with an anti-HA antibody. Actin was used as a control. (H) The UPB1 protein phosphorylation status was tested in *35S*::*UPB1-HA-Flag*, *35S*::*UPB1-HA-Flag/bin2-1*(+/-), and *35S*::*UPB1-HA-Flag/bin2-3bil1bil2* seedlings. UPB1 was detected with an anti-HA antibody, and separated on an SDS/PAGE gel containing Phos-tag reagent.

To detect whether phosphorylation of UPB1 by BIN2 can affect UPB1 protein stability, we assessed the stability of UPB1 with or without BL or bikinin treatment in the presence of MG132 and cycloheximide (CHX) treatment (Fig 4A). It showed that UPB1 was slightly reduced under the CHX treatment, and BL/bikinin accelerated the degradation of UPB1. MG132 inhibited the protein degradation of UPB1 in the presence of BL or bikinin. The UPB1 protein level in BL+CHX+MG132 and Bikinin+CHX+MG132-treated plants was almost same as the control treatment. These result suggest that the function of UPB1 in BR signaling can be regulated at the protein level via the 26S proteasome pathway.

To explore whether BIN2 could functionally depend on UPB1 in regulating root meristem development, we crossed the *35S*::*UPB1-HA-Flag* transgenic line with the *bin2-1*(+/-) mutant to generate *35S*::*UPB1-HA-Flag/bin2-1*(+/-) and *35S*::*UPB1-HA-Flag/bin2-1*(+/+) plants (Fig 4B). These plants showed substantially reduced primary root length, meristem size, and cell number compared with those of control (Fig 4C–4F). In addition, we obtained *35S*::*UPB1-HA-Flag* transgenic lines on a *bin2-3bil1bil2* background, and the primary root length, meristem size, and cell number of *35S*::*UPB1-HA-Flag/bin2-3bil1bil2* were significantly increased compared with those of *35S*::*UPB1-HA-Flag*, demonstrating that BIN2 is critical for UPB1 biological function. These data showed that BIN2 functionally interacts with UPB1 in common genetic pathway to control root meristem development.

Moreover, the *35S*::*UPB1-HA-Flag/bin2-1*(+/-) seedlings was treated with CHX, BL, or bikinin, and the UPB1 protein level was slightly reduced compared with that of the control under different treatments, suggesting BRs promote UPB1 degradation though the inhibition of BIN2 (Fig 4G). In addition, we tested the phosphorylation status of the UPB1 protein on the *upb1-1*, *bin2-1*(+/-), and *bin2-3bil1bil2* backgrounds (Fig 4H). The phosphorylation levels of UPB1 were increased in *bin2-1*(+/-) and decreased in *bin2-3bil1bil2* compared with those on *upb1-1* mutant backgrounds. However, the expression level of *UPB1* was not significantly changed in these transgenic plants (S4 Fig). These results suggest that UPB1 acts genetically downstream of BIN2 in the BR response.

### Mutation of a conserved BIN2 phosphorylation consensus leads to weak UPB1 stabilization

**Fig 5:**
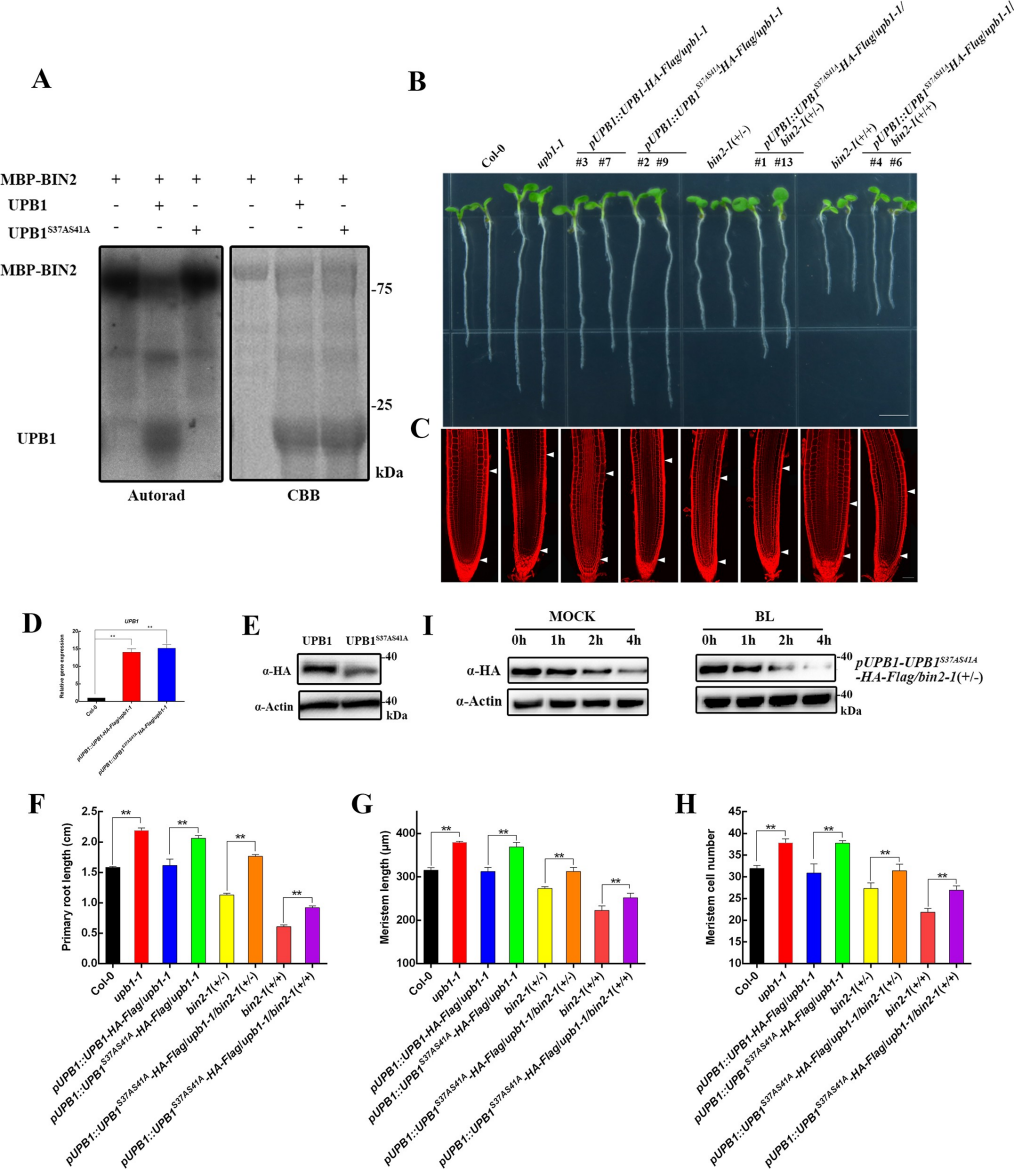
The Ser37 and Ser41 residues are critical for the functions of the UPB1 protein. (A) BIN2 phosphorylates UPB1 at the Ser37 and Ser41 residues. The left blot shows autoradiography. The right blot shows the CBB staining to indicate the loading of recombinant proteins. (B) Phenotypes of 5-day-old seedlings of Col-0, *upb1-1*, *pUPB1-UPB1-HA-Flag/upb1-1*, *pUPB1-UPB1*^*S37AS41A*^*-HA-Flag/upb1-1*, *bin2-1*(+/-), *pUPB1-UPB1*^*S37AS41A*^*-HA-Flag/upb1-1/bin2-1*(+/-), *bin2-1*(+/+), and *pUPB1-UPB1*^*S37AS41A*^*-HA-Flag/upb1-1/bin2-1*(+/+). Bar = 0.5 cm. (C) Root meristem of Col-0, *upb1-1*, *pUPB1-UPB1-HA-Flag/upb1-1*, *pUPB1-UPB1*^*S37AS41A*^*-HA-Flag/upb1-1*, *bin2-1*(+/-), *pUPB1-UPB1*^*S37AS41A*^*-HA-Flag/upb1-1/bin2-1*(+/-), *bin2-1*(+/+), and *pUPB1-UPB1*^*S37AS41A*^*-HA-Flag/upb1-1/bin2-1*(+/+) in 5-day-old seedlings. White arrowheads (below) mark the position of the quiescent center (QC), and white arrowheads (above) mark the end of the meristem where cells start to elongate. Bar = 50 μm. (D) Expression analysis of *UPB1* in the roots of *pUPB1-UPB1-HA-Flag/upb1-1* and *pUPB1-UPB1*^*S37AS41A*^*-HA-Flag/upb1-1* seedlings at 5 days old. The experiments were repeated three times with similar results. (E) Western blot detection of the UPB1 level in *pUPB1-UPB1-HA-Flag/upb1-1* and *pUPB1-UPB1*^*S37AS41A*^*-HA-Flag/upb1-1* seedlings. UPB1 was detected with an anti-HA antibody. Actin was used as a control. The primary root length (F), meristem size (G), and meristem cell number (H) of the seedlings shown in (B). Date means ± SD (n≥20). Double asterisk represent highly significant differences (**, P<0.01; Student’s *t* test). (I) *In vivo* UPB1 degradation assays. Ten-day-old *pUPB1-UPB1*^*S37AS41A*^*-HA-Flag/upb1-1/bin2-1*(+/-) transgenic seedlings was treated without or with 1 μM BL. Samples were collected at the indicated time points. UPB1 was detected with an anti-HA antibody. Actin was used as a control.

BIN2 phosphorylates serine and threonine residues in a short conserved motif (S/T)-X-X-X-(S/T) [23]. We identified only one consensus motif, S^37^VEAS^41^ that was conserved in the UPB1 protein, thus we performed site-directed mutagenesis of UPB1 and an *in vitro* kinase assay (Fig 5A). The phosphorylation of UPB1 was significantly reduced in UPB1^S37AS41A^ compared with that in the control, indicating that BIN2 mediated the phosphorylation of UPB1 was almost via the S37 and S41 sites. To explore the biological function of UPB1 phosphorylation, we transformed UPB1 and its mutated forms by its native promoter into the *upb1-1* mutant. The *pUPB1-UPB1-HA-Flag* completely rescued the root phenotype of the *upb1-1* mutant, and *pUPB1-UPB1*^*S37AS41A*^*-HA-Flag* did not rescued the phenotype of the *upb1-1* mutant (Fig 5B and 5C). Interestingly, the UPB1 protein level in *pUPB1-UPB1-HA-Flag/upb1-1* transgenic plants was significantly higher than that in *pUPB1-UPB1*^*S37AS41A*^*-HA-Flag/upb1-1* transgenic plants, while differences were not observed at the transcriptional level (Fig 5D and 5E), indicating that the phosphorylation sites S37 and S41 are important for the stability of the UPB1 protein. Next, we crossed *pUPB1-UPB1*^*S37AS41A*^*-HA-Flag/upb1-1* with the *bin2-1*(+/-) mutant, as shown in Fig 5B. Unlike normal UPB1, UPB1^S37AS41A^ overexpression on the *bin2-1*(+/-) background partially rescued the primary root length, meristem size, and meristem cell number (Fig 5F–5H). Moreover, the degradation rate of UPB1^S37AS41A^ was no significant change in the *pUPB1-UPB1*^*S37AS41A*^*-HA-Flag/upb1-1/bin2-1*(+/-) plants in the presence of BL at initial time course (1 h), and *bin2-1*(+/-) not enhanced the resistant of UPB1^S37AS41A^ after 2 h and 4 h of BL treatment (Fig 5I), demonstrating that BIN2 inhibits root meristem development by phosphorylating the S37 and S41 sites in UPB1. In addition, UPB1 is a non-cell-autonomous transcription factor, and S37 and S41 replacement may change its subcellular localization. UPB1-GFP and UPB1^S37AS41A^-GFP signals were mainly localized to the nucleus, suggesting that the S37 and S41 mutants did not change UPB1 localization (S5 Fig). Taken together, these data demonstrate that the conserved BIN2 phosphorylation residue is essential for UPB1 function in mediating root meristem growth.

### BIN2 increases the transcriptional activity of UPB1

To investigate whether BIN2 can increase the transcriptional activity of UPB1, we examined the transcription levels of three genes, *Peroxidase 39* (*Per39*), *Peroxidase 40* (*Per40*), and *Peroxidase 57* (*Per57*), which were well established as UPB1 target genes [20]. We fused promoters of these genes with the *luciferase* gene to generate reporter constructs. UPB1, BIN2, and UPB1 together with BIN2 were co-expressed with the reporter constructs in the *upb1-1* mutant protoplasts (S6A–S6C Fig). UPB1 repressed the expression of these reporter genes, and BIN2 alone did not affect reporter gene expression. However, these reporter gene expression levels were lower when UPB1 and BIN2 were co-expressed than when UPB1 was expressed alone. Further evidence supports the hypothesis that BIN2 increases the DNA-binding activity of UPB1 in ChIP assays. As shown in S6D–S6F Fig, the fold enrichment of *Per39*, *Per40*, and *Per57* was significantly increased in *35S*::*UPB1-HA-Flag/bin2-1*(+/-) compared to that in *35S*::*UPB1-HA-Flag*, and the UPB1 binding ability was not significantly increased by the addition of MG132. Taken together, these results demonstrate that BIN2 increases the transcriptional activity of UPB1 by phosphorylation.

### BES1 binding to the E-box element of the *UPB1* promoter

**Fig 6:**
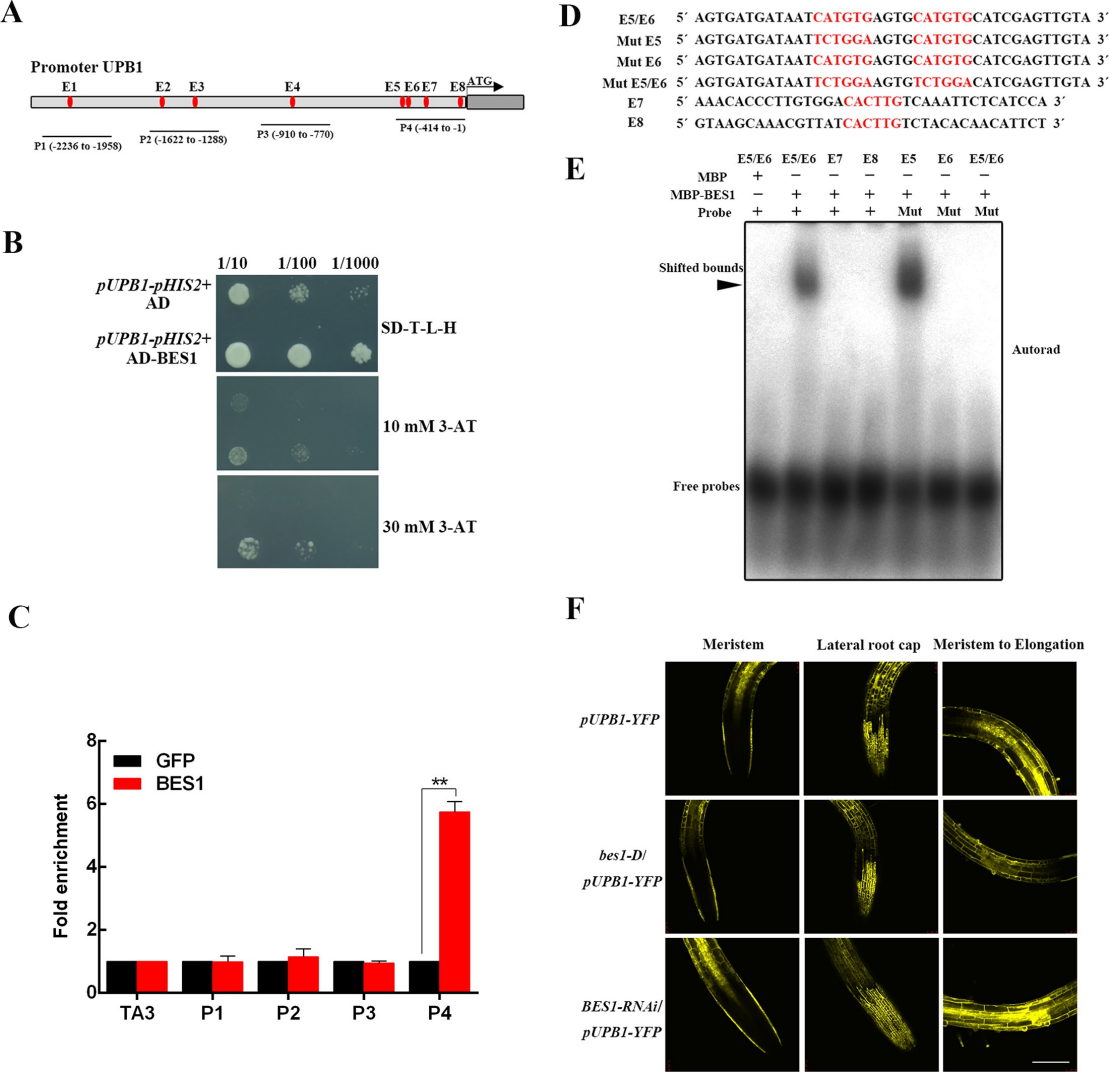
BES1 regulates UPB1 at the transcriptional level. (A) Diagram of the UPB1 promoter showing the relative positions of eight E-box (CANNTG) elements. The red plots represent the positions of the E-box elements. The lines show the positions of the primers used in ChIP-qPCR. (B) Y1H assays involving BES1 and the UPB1 promoter. (C) ChIP-qPCR assays of the *UPB1* promoter were performed using an anti-BES1 antibody. (D) Sequences of the probes used in the EMSAs. (E) EMSAs were conducted using recombinant MBP-BES1 protein purified from *E*. *coli* and synthesized biotinylated probes in the presence or absence of unlabeled wild-type or mutant probes. The arrow indicates the specific BES1/probe bands. (F) The expression of *pUPB1-YFP* in wild-type, *bes1-D*, and *BES1-RNAi* seedlings. Three zones with YFP signal were evaluated, including the meristem, lateral root map, and meristem to the elongation zone. Bar = 50 μm.

To investigate how BRs control *UPB1* gene expression, we examined the cis-acting elements of the *UPB1* promoter and found several E-boxes (CANNTG), constituting a BES1 affinity binding site (Fig 6A). Y1H assays revealed that BES1 targets the promoter of *UPB1* (Fig 6B). Moreover, ChIP assays revealed that BES1 could bind the *UPB1* promoter encompassing the P4 zone (Fig 6C). Four E-box cis-elements were found to exist in the BES1-binding fragments in P4 zone. DNA probes containing one or two E-box cis-element sequences were synthesized and tested with recombinant MBP-BES1 by EMSAs. MBP-BES1 was able to bind the DNA E6 probe but not the mutant probes in which the E-box was replaced with TCTGGA sequences (Fig 6D and 6E).

To obtain a further detailed understanding of the transcriptional regulation of *UPB1* expression by BES1, we crossed *bes1-D* and *BES1-RNAi* with *pUPB1-YFP* to generate *bes1-D/pUPB1-YFP* and *BES1-RNAi/pUPB1-YFP* mutants, respectively (Fig 6F). The fluorescence signal was strong in *BES1-RNAi/pUPB1-YFP* and weaker in *bes1-D/pUPB1-YFP* compared with that in the control. Moreover, we did not discover an interaction between UPB1 and BES1 (S7A and S7B Fig), and UPB1 overexpression or deficiency had little effect on the phosphorylation status of BES1 (S7C Fig). These results suggest that the transcription of *UPB1* is negatively regulated by BES1.

### UPB1 is a negative regulator of the PRE2/3 on regulate root meristem pathway

**Fig 7:**
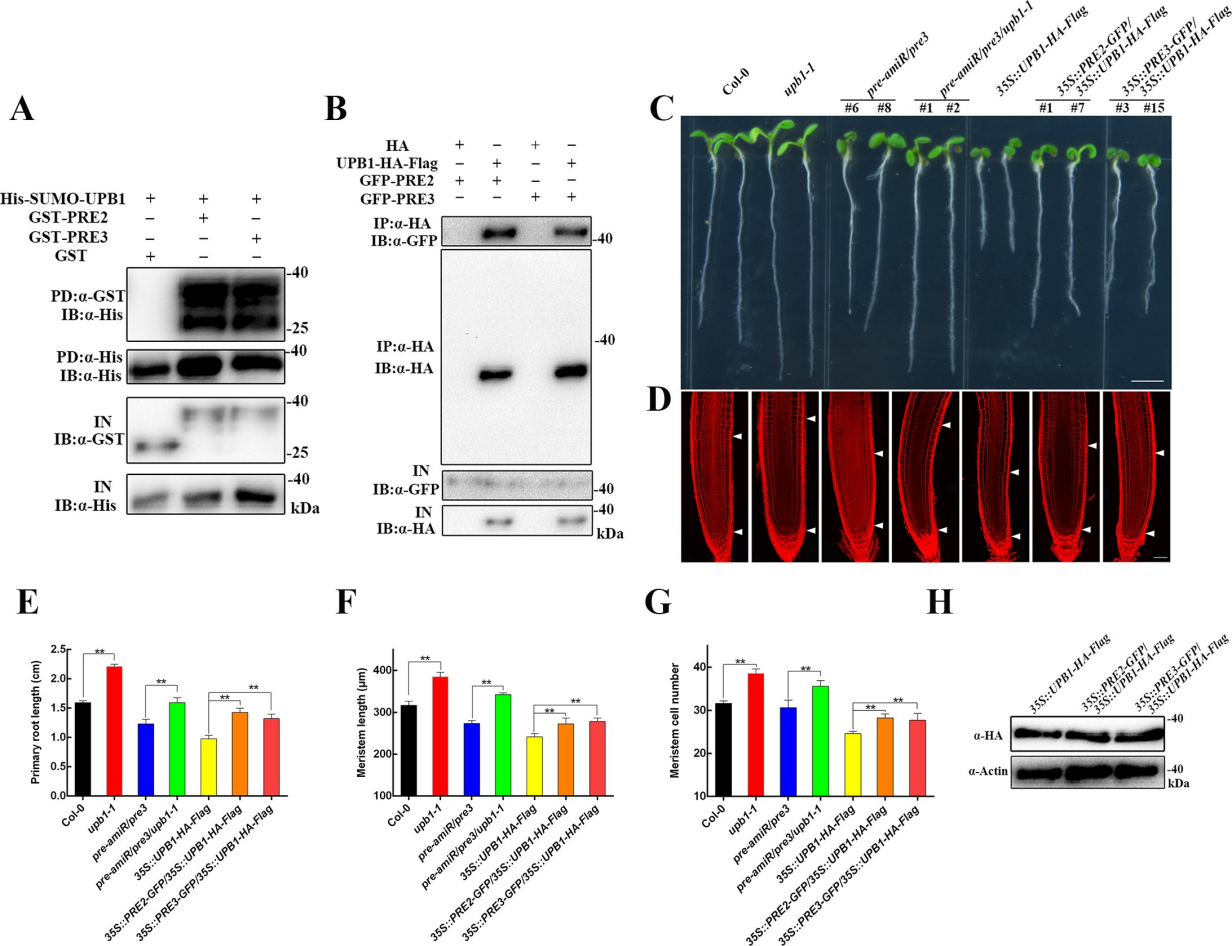
PRE2/3 interacts with UPB1. (A) His pull-down assays of PRE2/3 and UPB1. His-SUMO-UPB1, GST-PRE2, GST-PRE3, and GST were purified from *E*. *coli*. Purified proteins were used for the pull-down assay. His-SUMO-UPB1 protein was detected with an anti-His antibody, and the GST-PRE2, GST-PRE3, and GST proteins were detected with anti-GST antibodies. (B) Co-IP assays of UPB1 and PRE2/3. Protein extracts from 4-week-old *35S*::*PRE2-GFP/35S*::*HA-Flag*, *35S*::*PRE2-GFP/35S*::*UPB1-HA-Flag*, *35S*::*PRE3-GFP/35S*::*HA-Flag*, and *35S*::*PRE3-GFP/35S*::*UPB1-HA-Flag* seedlings, An anti-HA antibody was used for immunoprecipitation, and immunoblot analysis was performed with anti-HA and anti-GFP antibodies. (C) Phenotypes of 5-day-old seedlings of Col-0, *upb1-1*, *pre-amiR/pre3*, *pre-amiR/pre3/upb1-1*, *35S*::*UPB1-HA-Flag*, *35S*::*PRE2-GFP/35S*::*UPB1-HA-Flag*, and *35S*::*PRE3-GFP/35S*::*UPB1-HA-Flag*. Bar = 0.5 cm. (D) Root meristem of Col-0, *upb1-1*, *pre-amiR/pre3*, *pre-amiR/pre3/upb1-1*, *35S*::*UPB1-HA-Flag*, *35S*::*PRE2-GFP/35S*::*UPB1-HA-Flag*, and *35S*::*PRE3-GFP/35S*::*UPB1-HA-Flag* in 5-day-old seedlings. White arrowheads (below) mark the position of the quiescent center (QC), and white arrowheads (above) mark the end of the meristem where cells start to elongate. Bar = 50 μm. The primary root length (E), meristem size (F), and meristem cell number (G) in the seedlings shown in (C). Date means ± SD (n≥20). Double asterisk represent highly significant differences (**, P<0.01; Student’s *t* test). (H) The UPB1 protein levels were detected in *35S*::*UPB1-HA-Flag*, *35S*::*PRE2-GFP/35S*::*UPB1-HA-Flag*, and *35S*::*PRE3-GFP/35S*::*UPB1-HA-Flag* transgenic lines. UPB1 was detected with an anti-HA antibody. Actin was used as a control.

To identify novel components involved in UPB1-regulated root meristem development, we used the *pUPB1-UPB1-HA-Flag/upb1-1* transgenic plants followed by immunoprecipitation coupled to mass spectrometry (IP-MS) and detected high enrichment of the PRE3 peptide (encode a bHLH gene, *At1g74500*). Given that PRE3 has five homologous genes, we carried out a BiFC assay to detect UPB1-interacting proteins. Interestingly, we found that PRE2 also interact with UPB1 (S8 Fig). His pull-down assays showed that GST-PRE2 and GST-PRE3 could bind to His-SUMO-UPB1, suggesting that UPB1 interacts with PRE2/3 *in vitro* (Fig 7A). Moreover, we generated the overexpression lines *35S*::*PRE2-GFP* and *35S*::*PRE3-GFP* on the Col-0 background, and crossed them with *35S*::*UPB1-HA-Flag* and *35S*::*HA-Flag* plants, respectively. Co-IP analysis revealed that the *PRE2-GFP* and *PRE3-GFP* proteins were pulled down from *35S*::*PRE2-GFP/35S*::*UPB1-HA-Flag* and *35S*::*PRE3-GFP/35S*::*UPB1-HA-Flag* plants but not from control plants (Fig 7B). These results demonstrate that PRE2/3 interacts with the UPB1 protein *in vitro* and *in vivo*.

A previous study using the artificial microRNA method to generate a *pre-amiR* mutant (*PRE1/2/5/6* expression was significantly lower than that in the control) showed that dwarf phenotypes were similar to BR-deficient mutants [24]; however, the phenotype of the *pre3* mutant was not noticeably different from that of the wild-type. We crossed the *pre3* mutant with the *pre-amiR* mutant to generate *pre-amiR/pre3* mutant, which showed a shorter root and shorter meristem but normal cell number compared with those of the wild-type (S9A–S9F Fig). The expression levels of *PRE1/2/5/6* were reduced in *pre-amiR/pre3*, *PRE2/3* in *35S*::*PRE2-GFP* and *35S*::*PRE3-GFP* transgenic plants were dramatically increased than wild-type (S9G and S9H Fig) Moreover, the meristem cell size was significantly increased in *35S*::*PRE2-GFP* and *35S*::*PRE3-GFP* transgenic plants compared with that in the wild-type control. Interestingly, BRs induced *PRE2* and *PRE3* expression in roots (S10 Fig). To investigate the genetic crosstalk between PRE2/3 and UPB1, the *upb1-1* mutant was crossed with *pre-amiR/pre3* to obtain the *pre-amiR/pre3/upb1-1* mutant. The primary root length and meristem size of the *pre-amiR/pre3* mutant were obviously increased, and the root cell number was slightly increased by deficient UPB1 (Fig 7C–7G). Moreover, the *35S*::*PRE2-GFP* and *35S*::*PRE3-GFP* transgenic plants could increase the primary root length, meristem size, and meristem cell number of *35S*::*UPB1-HA-Flag* transgenic plants. Western blot analysis showed that the UPB1 protein levels in the *35S*::*PRE2-GFP/35S*::*UPB1-HA-Flag* and *35S*::*PRE3-GFP/35S*::*UPB1-HA-Flag* seedlings were not significantly different than those in *35S*::*UPB1-HA-Flag* seedlings (Fig 7H). This genetic evidence shows that UPB1 functions downstream of PRE2/3 to inhibit root meristem growth.

### PRE2/PRE3 represses the transcriptional activity of UPB1

A previous study showed that PRE3 localized in the nucleus and cytosol [25]. However, our research revealed that PRE2/3 interacts with UPB1 in the nucleus (S8 Fig), indicating that PRE3 is recruited by UPB1 from the cytoplasm to the nucleus. Interestingly, PRE2-GFP was also detected in the cytoplasm and nucleus (S11A Fig). We further examined whether PRE2/3 accumulation in the nucleus might be affected by UPB1. Relatively higher PRE2/3 expression in the nucleus was detected in the *35S*::*PRE2-GFP/35S*::*UPB1-HA-Flag* and *35S*::*PRE3-GFP/35S*::*UPB1-HA-Flag* seedlings compared with that in *35S*::*PRE2-GFP* and *35S*::*PRE3-GFP* seedlings, although the total levels were similar in PRE2/3 (S11B Fig). These results suggest that in the presence of UPB1, PRE2/3 is reduced in the cytoplasm and accumulates in the nucleus.

We next explored whether PRE2/3 influences the transcriptional activity of UPB1. UPB1, PRE2, PRE3, UPB1 together with PRE2, and UPB1 together with PRE3 were co-expressed with the reporter constructs (S12A–S12C Fig). PRE2/3 alone did not affect the expression of reporter genes, and the reporter activity in the PRE2/3 and UPB1 co-expression group was increased compared with that in the UPB1 alone group. Further evidence supported that PRE2/3 reduced the DNA binding activity of UPB1 in ChIP assays (S12D–S12F Fig). The fold enrichment of UPB1 on *Per39*, *Per40*, and *Per57* promoters was significantly reduced in *35S*::*PRE2-GFP/35S*::*UPB1-HA-Flag* and *35S*::*PRE3-GFP/35S*::*UPB1-HA-Flag* compared with that in *35S*::*UPB1-HA-Flag*. Taken together, these results demonstrate that PRE2/3 negatively regulates the transcriptional activity of UPB1.

### BIN2 inhibits the interaction between PRE2/3 and UPB1

**Fig 8:**
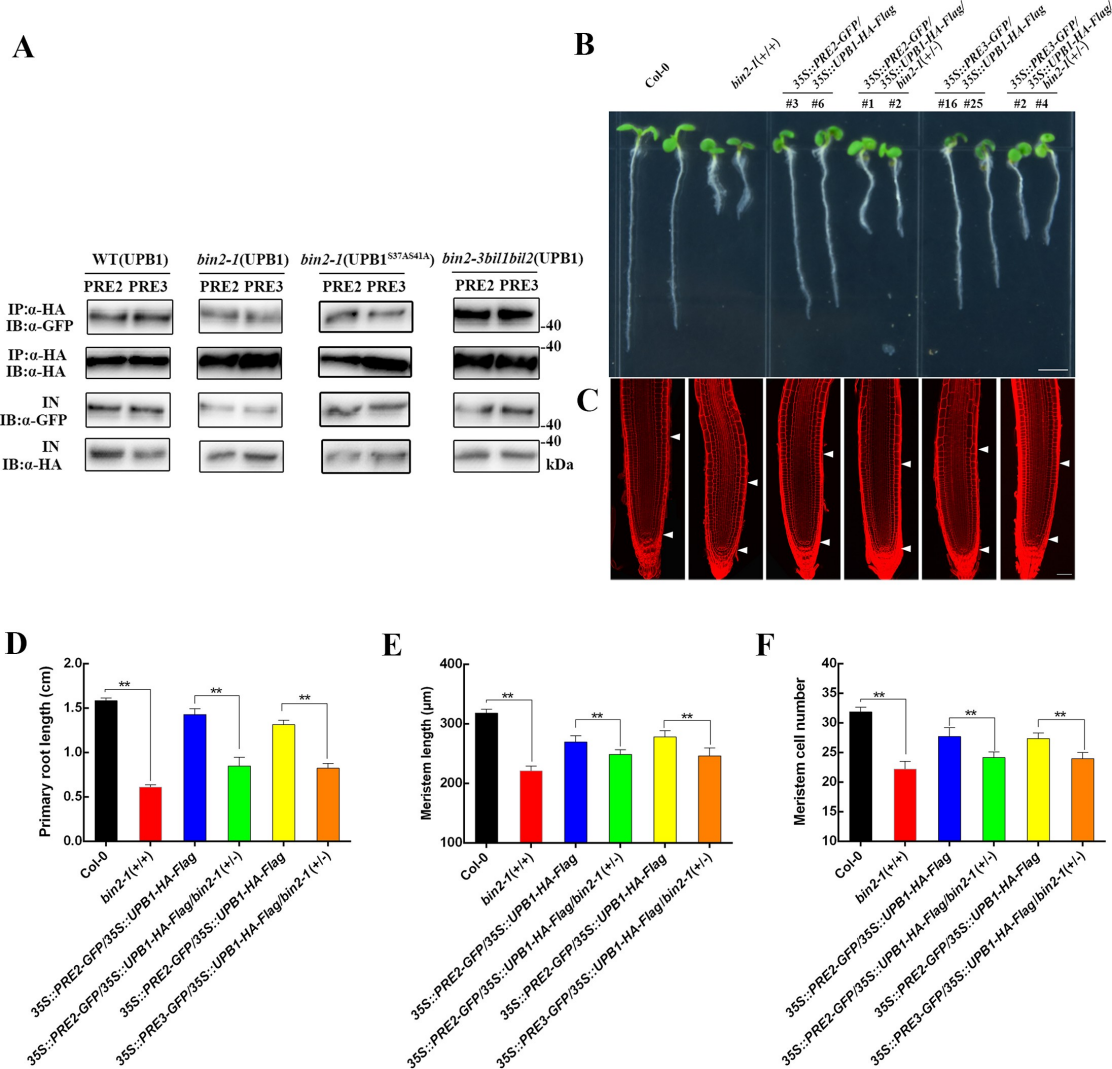
BIN2 affects the interactions between UPB1 and PRE2/3. (A) BIN2 influences the interaction between UPB1 and PRE2/3 in *Arabidopsis*. 35S::UPB1-HA-Flag together with 35S::PRE2-GFP, 35S::UPB1-HA-Flag together with 35S::PRE3-GFP, 35S::UPB1^S37AS41A^-HA-Flag together with 35S::PRE2-GFP, and 35S::UPB1^S37AS41A^-HA-Flag together with 35S::PRE3-GFP plasmids were co-transformed into Col-0, *bin2-1*(+/-), and *bin2-3bil1bil2* seedling protoplasts, respectively. An anti-HA antibody was used for immunoprecipitation, and immunoblot analysis was performed with anti-HA and anti-GFP antibodies. (B) Phenotypes of 5-day-old seedlings of Col-0, *bin2-1*(+/+), *35S*::*PRE2-GFP/35S*::*UPB1-HA-Flag*, *35S*::*PRE2-GFP/35S*::*UPB1-HA-Flag*/*bin2-1*(+/-), *35S*::*PRE3-GFP/35S*::*UPB1-HA-Flag*, and *35S*::*PRE3-GFP/35S*::*UPB1-HA-Flag*/*bin2-1*(+/-). Bar = 0.5 cm. (C) Root meristem of Col-0, *bin2-1*(+/+), *35S*::*PRE2-GFP/35S*::*UPB1-HA-Flag*, *35S*::*PRE2-GFP/35S*::*UPB1-HA-Flag/bin2-1*(+/-), *35S*::*PRE3-GFP/35S*::*UPB1-HA-Flag*, and *35S*::*PRE3-GFP/35S*::*UPB1-HA-Flag/bin2-1*(+/-) in 5-day-old seedlings. White arrowheads (below) mark the position of the quiescent center (QC), and white arrowheads (above) mark the end of the meristem where cells start to elongate. Bar = 50 μm. The primary root length (D), meristem size (E), and meristem cell number (F) of the seedlings shown in (B). Date means ± SD (n≥20). Double asterisk represent highly significant differences (**, P<0.01; Student’s *t* test).

To study the effect of BIN2 on PRE2/3-UPB1 complex formation, we co-expressed UPB1 and PRE2, UPB1 and PRE3, UPB1^S37AS41A^ and PRE2, and UPB1^S37AS41A^ and PRE3 in Col-0, *bin2-1*(+/-), and *bin2-3bil1bil2* seedlings protoplasts (Fig 8A). Their interactions were dramatically reduced in *bin2-1*(+/-) and obviously increased in *bin2-3bil1bil2* seedlings compared with those in Col-0. The interaction between UPB1^WT^ and PRE2/3 were dramatically reduced in *bin2-1*(+/-) and obviously increased in *bin2-3bil1bil2* seedlings compared with those in Col-0. Interesting, the interaction between UPB1^S37AS41A^ and PRE2/3 was significantly enhanced compared with UPB1-PRE2/3 interaction in *bin2-1*(+/-) plants, suggesting that BIN2 phosphorylation may influence the interaction between UPB1 and PRE2/3. Moreover, we crossed *bin2-1*(+/-) with *35S*::*PRE2-GFP/35S*::*UPB1-HA-Flag* and *35S*::*PRE3-GFP/35S*::*UPB1-HA-Flag* seedlings to obtain the *35S*::*PRE2-GFP/35S*::*UPB1-HA-Flag/bin2-1*(+/-) and *35S*::*PRE3-GFP/35S*::*UPB1-HA-Flag/bin2-1*(+/-) plants (Fig 8B). The primary root length, meristem size, and cell number of *35S*::*PRE2-GFP/35S*::*UPB1-HA-Flag* and *35S*::*PRE3-GFP/35S*::*UPB1-HA-Flag* were partially suppressed by *bin2-1*(+/+) (Fig 8C–8F). Moreover, the expression levels of *PRE2* and *PRE3* were no significant changes in wild-type and *bin2-1*(+/+) plants (S13 Fig). We therefore conclude that the BIN2 attenuates the interaction of UPB1 with PRE2/3 and leads to increased activation of UPB1 to inhibit root meristem development.

## Discussion

Recent studies have revealed that BR-mediated optimal root growth is controlled by antagonistic or synergistic interaction with auxin, ethylene, and ROS [14, 26]. However, the key components involved in BR-mediated root meristem development have been poorly studied. In this study, we showed that BIN2 interacted with and phosphorylated UPB1 to regulate root meristem, and BES1 could directly bind to the promoter of *UPB1* and repress its expression. Genetic analyses suggested that UPB1 functions as a downstream component of BR signaling in regulating root meristem. In addition, our results demonstrated that BIN2 played a role in interfering with the interaction between UPB1 and PRE2/3 through phosphorylation. Thus, our finding defined an important genetic and molecular mechanism for BRs in regulating root meristem growth and development.

The root meristem size in BR biosynthetic and responsive mutants was significantly altered, demonstrating that BIN2 and BES1/BZR1 were involved in root meristem development (Fig 1). Consistent with a previous study which reported BES1/BZR1 could bind in the promoters of *ACS7*, *ACS9*, and *ACS11* to regulate root development [26]. Interestingly, some researches showed BRs could regulate root hair development by BIN2 and/or its upstream components, and BRs also modulated control lateral root growth by BSU1 or its upstream components [27, 28]. Taken together, these results suggested that BRs modulated root development in different sections through distinct molecular processes. Future research should explore how BR coordinate growth balance among primary root, lateral root, and root meristem in plant developmental processes.

BIN2 is a key component mediating BR signaling during root development. Although this phenomenon of short-root is first observed in *bin2-1*(+/-) mutant [29], the molecular mechanism by which BIN2 regulated root development remains unclear. We found that the transcription factor UPB1 was a substrate of BIN2, and that BIN2 facilitated the stabilization and promoted the transcriptional activity of UPB1 through phosphorylation (Figs 2 and 4). In accordance with this finding, *35S*::*UPB1-HA-Flag/bin2-1*(+/-) and *35S*::*UPB1-HA-Flag/bin2-1*(+/+) plants showed enhanced the BIN2 function in regulating root meristem development (Fig 4B). Notably, our experiments also indicated that BIN2 could stabilize the UPB1 protein in response to BR treatment (Fig 4G). Accordant BRs depends on BIN2 phosphorylating TTG1 to inhibit the WER-GL3/EGL3-TTG1 complex during root hair patterning [28]. However, BRs play a minor role in the BIN2-mediated activation of ARF7 and ARF19 in mediating lateral root development [27]. These results demonstrated that function of BIN2 in mediating root development is very complicated in plants. Moreover, our experiments also indicated the *UPB1* expression was regulated via the transcription factor BES1 (Fig 6). Genetic analyses showed that the lack of *UPB1* partially rescued the short-root phenotype in BR-deficient mutants, suggesting that *UPB1* played a negative role in the BR signaling pathway (Fig 3). Therefore, the strict regulation of BIN2 kinase activity was crucial for BR regulate root meristem development.

Another interesting phenomenon was that BR could induce the expression of *PRE2/3* (S10 Fig). PRE2/3 interacted with UPB1 and this interaction inhibited the binding affinity of UPB1 to *Per* genes (Figs 6 and S12), which was consistent with the previous finding that PRE3 interacted with AIF1 and repressed its function [25]. PRE1 could interact with three AIF but not with AIF1 [30]; however, we did not detect an interaction between UPB1 and PRE1 (S8 Fig). AIF1 and AIF2 also were identified as BIN2 substrates in developmental processes [25, 31]. Thus, we concluded that AIFs and UPB1 functioned overlaps in some details on growth and developmental processes. In addition, genetic evidence indicated that BIN2 phosphorylating UPB1 to promote dissociation between UPB1 and PRE2/3 (Fig 8), indicating that BIN2 played an additional regulatory role in BR signaling by regulating the hetero-dimerization of UPB1 with PRE2/3. This finding also provided direct evidence that PRE3 overexpression partially recovered the dwarf phenotype of *bin2-1*(+/-) but worked not good in *bin2-1*(+/+) [25]. Moreover, our results showed that BIN2 phosphorylated UPB1 at two conserved Ser residues (Fig 5), and BIN2 inhibited the interaction between PRE2/3 and UPB1, and Ser37 and Ser41 mediates this reduction (Fig 8). In accordance with BIN2 influenced the interaction between INDUCE OF CBF EXPRESSION 1 (ICE1) and the E3 ligase HIGH EXPRESSION OF OSMOTICALLY RESPONSIVE GENE 1 (HOS1) mainly through conserved Ser94 in ICE1 [32]. In addition, BR-induced transcriptional repression and protein degradation negatively regulate UPB1, reinforcing the BZR1/BES1-mediated positive BR signaling pathway (Fig 6). It would be interesting to explore the subtle relationship between BIN2 and these bHLH transcription factors by generating multiple layers crosstalk in regulating root meristem development. This would require substantial effort, as both BES1 and PRE have several homologs that function redundantly [7, 24].

**Fig 9:**
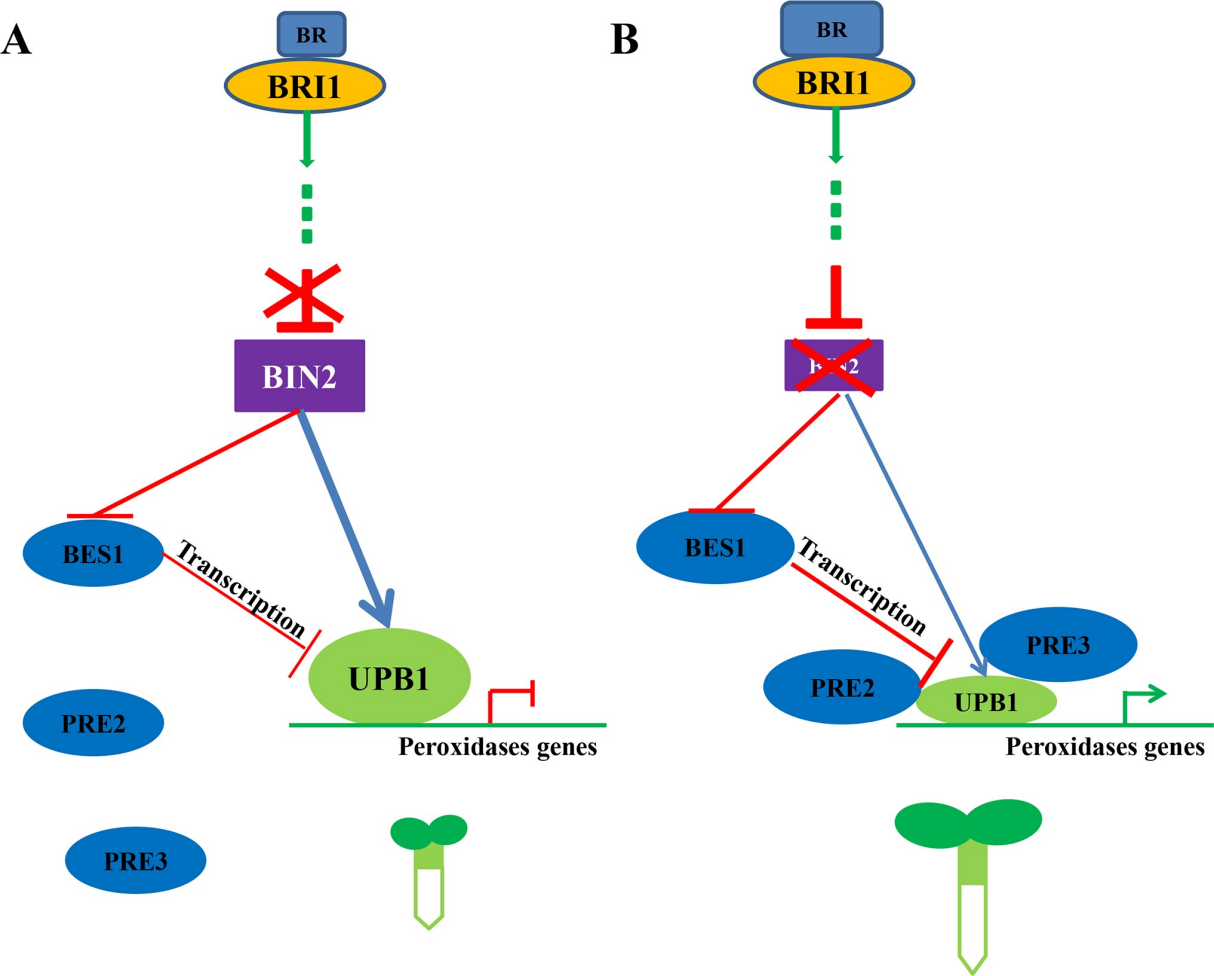
A hypothetical model illustrating how BIN2 acts with UPB1 to regulate transcription and root meristem development in *Arabidopsis*. (A) In the absence of BRs, the activated BIN2 phosphorylates UPB1, leading to improved stability and transcriptional activity of UPB1. Furthermore, BIN2 represses the interaction between PRE2/3 and UPB1, resulting in UPB1 repressing the expression of peroxidases, thereby reducing root meristem growth. (B) In the presence of BRs, the BIN2 kinase activity is repressed, leading to the reduced phosphorylation of UPB1. Moreover, PRE2/3 is induced and PRE2/3 protein accumulates in the nucleus, where it interacts with UPB1 and consequently represses its transcriptional activity of UPB1, thereby promoting root meristem growth.

Taken together, these results propose a regulatory model to show how BR signaling regulates BIN2-UPB1 protein complex assembly and activity in modulating root meristem development. In the absence of BRs (Fig 9A), BIN2 phosphorylates UPB1 and stabilizes the protein, subsequently blocking the interaction between the UPB1 and PRE2/3 proteins, resulting in enhanced DNA-binding capacity and inhibiting root meristem development. In the presence of BRs (Fig 9B), BIN2 kinase activity is repressed by BR, and UPB1 is not stabilized and degraded by the 26S proteasome. PRE2 and PRE3 relocates from the cytoplasm to the nucleus and interacts with UPB1, consequently strengthening the signaling output effect of BR on root meristem development. Collectively, we conclude that BR-regulated root meristem size is mediated by the BIN2-UPB1 module.

## Materials and methods

### Plant materials and growth conditions

The *Arabidopsis* mutant *pre3* (N558700) and *pUPB1-YFP* (N2106126) were purchased from *The European Arabidopsis Stock Centre* (NASC). The following mutants were previously reported: *det2-1*, *bri1-5*, *bin2-1*, *bin2-3bil1bil2*, *BES1-RNAi*, and *bes1-D* [4], *pre-amiRNA* [24], and *upb1-1* [20], *bri1-5* and *bin2-3bil1bil2* are in Ws-2 background, and other plants are in Col-0 background. All double and multiple mutants were from crossing and confirmed by RT-PCR or enzyme digestion analysis. *Arabidopsis* seeds were sterilized with 75% (v/v) ethanol and 0.1% Triton X-100 for approximately 20 min. Next, the seeds were washed with water three times, sprinkled and grown on 1/2 Murashige and Skoog (MS) plates, and stored at 4°C for 2 days in the dark, then culture in constant light culture under long-day conditions (16 h light/8 h dark) at 22°C.

### Plasmid construction and generation of transgenic plants

The full-length coding sequences of *UPB1*, *BIN2*, *PRE2*, and *PRE3* were cloned into the *pCAMBIA 1307-HA-Flag*, *pCAMBIA 1307-Myc*, or *pCAMBIA 1302-eGFP* vector. To obtain the *pUPB1-UPB1-HA-Flag/upb1-1* and *pUPB1-UPB1*^*S37AS41A*^*-HA-Flag/upb1-1* transgenic lines, a genomic fragment including the 3-kb promoter region of the *UPB1* and *UPB1* or *UPB1*^*S37AS41A*^ coding sequences was cloned into the *pCAMBIA 1307-HA-Flag* vector. These constructs were introduced into Col-0 or *upb1-1* mutants by *Agrobacterium tumefaciens*-mediated floral transformation. T3 transgenic plants were used for analysis.

### RNA extraction and qRT-PCR

Total RNA was extracted using the RNeasy Plant Mini Kit (Qiagen), and first-strand cDNA was synthesized from 1 μg of total RNA using the Transcription First Strand cDNA Synthesis Kits (Promega). Quantitative PCR analyses were performed using CFX (Bio-Rad) real-time PCR equipment with SYBR reagent. *ACTIN2* was used as an internal control. Primer sequences are listed in S1 Table.

### Immunoprecipitation and mass spectrometry (IP-MS)

IP-MS analyses were performed as previously described [33]. In brief, we mechanically ground four-week-old *pUPB1-UPB1-HA-Flag/upb1-1* plants in liquid nitrogen. Ten grams of powered tissue was resuspended in IP buffer, rotated for approximately 30 min at 4°C, and centrifuged, the supernatant was then incubated with HA agarose beads (Sigma), centrifuged, washed, and mixed with 20 μl of protein loading buffer. Then, the protein was separated by SDS-PAGE, stained with Coomassie blue and in-gel digested with trypsin. The digested peptide mixtures were analyzed by reverse-phase LC-MS/MS (Thermo Scientific Q Exactive HF-X).

### Transient transcription assay

*Arabidopsis* mesophyll cell protoplasts were prepared and transformed as described previously [34]. For luciferase assays, we cloned promoters of *At4g11290*, *At4g16270*, and *At5g17820* into the *pGreen II 0800* vector. Plasmids were singly or co-transformed into *Arabidopsis* protoplasts. Luciferase activities were measured using a luciferase assay system (Promega) after 16 h, and the data were normalized to REN activity. The experiments were repeated three times with similar results.

### Bimolecular fluorescence complementation analysis (BiFC)

For BiFC assays, the coding sequences of *BIN2*, *BIL1*, *BIL2*, *BES1*, and *PRE1/2/3/4/5/6* were cloned into the *pXY103* vector (nYFP). *UPB1* was cloned into the *pXY104* (cYFP) vector. These plasmids were introduced into *A*. *tumefaciens* (strain GV3101) and then transformed into young leaves of *N*. *benthamiana*. YFP fluorescence was analyzed using a fluorescence microscope (Lecia). The experiments were repeated three times with similar results.

### Electrophoretic mobility shift assay (EMSAs)

EMSAs assays were performed as previously described [35]. Briefly, approximately 1 ng of probe (10 000 cpm) with purified protein from *E*. *coli* was incubated with 20 μl of binding buffer (25 mM HEPES-KOH, pH 8.0, 50 mM KCl, 1 mM dithiothreitol and 10% glycerol) and labeled with ^32^P-γ-ATP. The reaction lasted for at least 30 min on ice and was resolved on 5% native polyacrylamide gels with TBE buffer (1.08 g/L Tris, 5.5 g/L boric acid, EDTA, pH 8.3).

### Phenotype and confocal microscopy analysis

Seedlings were grown on 1/2 MS medium for 5 days in the vertical position, and then treated with DMSO, various concentrations of BL, or various concentrations of BRZ for 24 h under long-day conditions (16 h light/8 h dark) at 22°C. For root length measurements, ImageJ software was used to measure the scanned pictures from digital images. Root meristem length and cell number were analyzed using the modified pseudo-schiff propidium iodide (mPS-PI) staining method [36]. Briefly, *Arabidopsis* seedlings were fixed in fixative (50% methanol and 10% acetic acid) at 4°C for 12 h. Next, tissue was rinsed to water for three times and transferred to 1% periodic acid for approximately 40 min at room temperature. The seedlings was rinsed again with water and incubated in Schiff reagent with propidium iodide (100 μg/mL propidium iodide, 0.15 N HCl, and 100 mM sodium metabisulphite) for approximately 1 h. Seedlings were then transferred onto microscope slides and covered with a chloral hydrate solution (1 mL glycerol, 4 g chloral hydrate, and 2 mL water). And then incubated overnight at room temperature. Hoyer’s solution (20 g glycerol, 200 g chloral hydrate, 30 g gum arabic, and 50 mL water) was replaced with chloral hydrate, and a cover slip was placed on top. The phenotype was determined with a laser scanning confocal microscope (Leica), and the PI signal was visualized at an emission wavelength ranging from 610 nm to 630 nm. YFP was observed from 520 nm to 540 nm. Twenty seedlings were taken and analyzed for each treatment and phenotype (S1 and S2 Data).

### *In vitro* pull-down assay

The coding sequence of *UPB1* was cloned into *pET32-His-SUMO* to construct *His-SUMO-UPB1*. Similarly, *BIN2* was cloned into *pETMALc-H* (*MBP*), and *PRE2* and *PRE3* were cloned into the *pGEX-4T-1* vector. The recombinant proteins were purified according to the manufacturer’s protocol. To test whether UPB1 interacts with BIN2, PRE2 and PRE3, approximately 30 μg of the His-SUMO-UPB1 protein was incubated with 30 μg of the MBP-BIN2, GST-PRE2, or GST-PRE3 protein in 400 μl of His pull-down binding buffer (20 mM Tris-HCl pH 8.0, 150 mM NaCl, 0.2% Triton). The reaction mixture was slowly shaken for approximately 3 h at 4°C. The beads were washed five times and protein loading buffer was added for 10 min at 100°C. Proteins were separated by 12% SDS-PAGE and tested with anti-His, anti-MBP, and anti-GST antibodies.

### Yeast one-hybrid (Y1H) and two-hybrid (Y2H) assays

Y1H assays was performed as previously described [37]. Briefly, the promoter region of *UPB1* was cloned into the reporter plasmid *pHIS2* and *BES1* gene was cloned into the effector plasmid *pAD-GAL4*. The effector and the reporter vectors were transformed into yeast cells (Y187) and growth on SD/-Trp-Leu-His plate or the plate with 3-amino-1,2,4-triazole (3-AT). The *pUPB1-pHIS2* vector and the *pAD-GAL4* plasmid were used as a negative control. Y2H assays were performed using the Matchmaker *GAL4* two-hybrid system (Clontech) [38]. The *UPB1* gene was cloned into the *pGADT7* vector, *BIN2*, *BIL1*, *BIL2*, and *BES1* were cloned into the *pGBKT7* vector. The constructs were transformed into yeast strain AH109 by LiCl/NaNc. Transformants were grown on SD/-Leu-Trp-His dropout plates.

### Yeast two-hybrid screen

The Mate & Plate Library-Universal Arabidopsis (Normalized, Clontech, 630487) was cloned into the *pACT* vector, and the *BIN2-pGBKT7* and prey plasmids or library DNA were co-transformed into the yeast strain AH109. Approximately 2x 10^7^ cell transformants were screened each time to select colonies that were grown on SD-H medium containing 3-AT (50 mM). The prey plasmid DNAs were isolated from yeast cells, and DNAs were extracted from the yeast plasmid extraction kit for sequence analyses (Tiangen).

### Coimmunoprecipitation (Co-IP) assays

The Co-IP assays were performed as previously described [39]. Total proteins from *35S*::*BIN2-Myc/35S*::*UPB1-HA-Flag*, *35S*::*BIN2-Myc/35S*::*HA-Flag*, *35S*::*PRE2-GFP/35S*::*UPB1-HA-Flag*, *35S*::*PRE3-GFP/35S*::*UPB1-HA-Flag*, *35S*::*PRE2-GFP/35S*::*HA-Flag*, and *35S*::*PRE3-GFP/35S*::*HA-Flag* seedlings were isolated with IP buffer and incubated with HA agarose beads (Sigma). The beads were detected by anti-HA (Sangon), anti-Myc (Abmart), and anti-GFP (Abmart) antibodies.

The *35S*::*UPB1-HA-Flag* and *35S*::*PRE2-GFP*, *35S*::*UPB1*^*S37AS41A*^*-HA-Flag* and *35S*::*PRE2-GFP*, *35S*::*UPB1-HA-Flag* and *35S*::*PRE3-GFP*, and *35S*::*UPB1*^*S37AS41A*^*-HA-Flag* and *35S*::*PRE3-GFP* plasmids were co-transformed into *Arabidopsis* protoplasts of Col-0, *bin2-1*, and *bin2-3bil1bil2* mutants. Total proteins were isolated with IP buffer incubated with anti-HA agarose beads. The beads were detected by anti-HA and anti-GFP antibodies.

### Phosphorylation assays

For *in vitro* phosphorylation assays, *His-SUMO-UPB1* and *His-SUMO-UPB1*^*S37AS41A*^ were removed from the 6x His tag using the TAGZyme Kit (Qiagen) and incubated with *MBP-BIN2* proteins in 15 μl of kinase reaction buffer (0.5 M Tris-HCl pH 7.5, 100 mM CaCl_2_, 25 mM MgCl_2_, 10 mM ATP, 100 mM NaVO_3_, 100 mM DTT) and 10 μCi ^32^P-γ-ATP. After incubation at 37°C for approximately 40 min, and 5 μl of protein loading buffer was added. Proteins were resolved by SDS-PAGE, and phosphorylation was detected by X-ray film (Typhoon 9410, Amersham). For *in vivo* phosphorylation assays, the UPB1 protein was immunoprecipitated from *35S*::*UPB1-HA-Flag*, *35S*::*UPB1-HA-Flag/bin2-1*(+/-), and *35S*::*UPB1-HA-Flag/bin2-3bil1bil2* transgenic plants. Proteins were separated by 12% SDS-PAGE with Phostag reagent (NARD Institute) and tested with an anti-HA antibody.

### Chromatin immunoprecipitation assays

ChIP assays were performed as previously described [40]. Briefly, four-week-old Col-0, *35S*::*UPB1-HA-Flag*, *35S*::*UPB1-HA-Flag/bin2-1*(+/-), *35S*::*PRE2-GFP/35S*::*UPB1-HA-Flag*, and *35S*::*PRE3-GFP/35S*::*UPB1-HA-Flag* transgenic seedlings were crosslinked with formaldehyde and the reaction was stopped by 125 mM glycine. Chromatin was sonicated to produce approximately 0.3 kb DNA fragments. The sonicated protein-DNA complexes were precipitated with an anti-HA antibody. After incubation with protein A beads, the beads were further washed with low-salt and high-salt buffer and reverse crosslinked with 200 mM NaCl. After removing proteins with proteinase K, DNA fragments were purified by phenol-chloroform extraction and ethanol precipitation. The DNA fragment was dissolved in TE buffer (10 mM Tris-HCl pH 8.0, 1 mM EDTA) and used as qPCR templates on the real-time system. The TA3 fragment served as the normalization control for qPCR analysis. The experiments were repeated three times with similar results.

### Nuclear fractionation

Nuclear fractionation was performed as previously described [41]. Fourteen-day-old seedlings were collected and homogenized in extraction buffer. Total protein extracts were filtered through three layers of Miracloth. After centrifugation at 4°C for 10 min, the pellet was washed twice with nuclei resuspension buffer (20 mM Tris-HCl pH 7.4, 2.5 mM MgCl_2_, 0.2% Triton X-100, and 25% (v/v) glycerol), and then used in Western blot analyses.

### Protein extraction and immunoblot analysis

Total protein was extracted with extraction buffer and centrifuged at 12,000 rpm for 10 min. The supernatant and sediment were discarded. For immunoblot analysis, total proteins were separated on 12% SDS-PAGE and detected by antibodies with enhanced chemiluminescence.

### Accession numbers

The Arabidopsis Genome Initiative identifiers for the genes described in this article are as follows: *BIN2* (*At4g18710*), *BIL1* (*At2g30980*), *BIL2* (*At1g06390*), *BES1* (*At1g19350*), *DET2* (*At2g38050*), *BRI1* (*At4g39400*), *UPB1* (*At2g47270*), *PER39* (*At4g11290*), *PER40* (*At4g16270*), *PER57* (*At5g17820*), *PRE1* (*At5g39860*), *PRE2* (*At5g15160*), *PRE3* (*At1g74500*), *PRE4* (*At3g47710*), *PRE5* (*At3g28857*), *PRE6* (*At1g26945*), and *BAS1* (*At2g26710*).

## Supporting information

S1 Fig BIL1/2 dose not interacts with UPB1 in BiFC and Y2H assays. (A) In BiFC assays. nYFP and cYFP, BIL1-nYFP and cYFP, BIL2-nYFP and cYFP, nYFP and UPB1-cYFP, BIL1-nYFP and UPB1-cYFP, and BIL2-nYFP and UPB1-cYFP were co-transformed in *N*. *benthamiana* leaves cells. Bar = 50 μm. (B) Y2H assays of the interaction between BIL1/2 and UPB1. Transformed yeast cells were grown on the SD-L-W or SD-L-W-H medium.
(TIF)

S2 Fig UPB1-overexpressing plants display constitutive BR-deficient phenotypes. (A) The expression profiles of *UPB1* in *Arabidopsis*. Bar = 50 μm. (B) Phenotypes of 5-day-old seedlings of Col-0, *upb1-1*, *35S*::*UPB1-HA-Flag#3*, and *35S*::*UPB1-HA-Flag#8*. Bar = 0.5 cm. (C) Root meristem of Col-0, *upb1-1*, *35S*::*UPB1-HA-Flag#3*, and *35S*::*UPB1-HA-Flag#8* in 5-day-old seedlings. White arrowheads (below) mark the position of the quiescent center (QC), and white arrowheads (above) mark the end of the meristem where cells start to elongate. Bar = 50 μm. The primary root length (D), meristem size (E), and meristem cell number (F) of the seedlings shown in (B). Date means ± SD (n≥20). Double asterisk represent highly significant differences (**, P<0.01; Student’s *t* test). (G) Expression analysis of *UPB1* in the roots of Col-0, *35S*::*UPB1-HA-Flag#3*, and *35S*::*UPB1-HA-Flag#8* seedlings at 5 days old. (H) Phenotypes of 4-week-old seedlings of Col-0, *upb1-1*, *35S*::*UPB1-HA-Flag#3*, and *35S*::*UPB1-HA-Flag#8*. Bar = 1 cm.
(TIF)

S3 Fig Expression of *UPB1* is down-regulation by BL. (A) *pUPB1-YFP* transgenic seedlings was absence or presence of 100 nM BL, and the fluorescence signal was detected at 0 h, 1 h, 2 h, and 4 h. Bar = 50 μm. (B) The expression of *UPB1* was examined by RT-qPCR in the roots of Col-0, *det2-1*, *bin2-1*(+/-), *bin2-1*(+/+), *BES1-RNAi*, WS, *bri1-5*, and *bin2-3bil1bil2* seedlings at 5 days old. (C) Expression analysis of *BAS1* in the roots of Col-0, *upb1-1*, *35S*::*UPB1-HA-Flag#3*, and *35S*::*UPB1-HA-Flag#8* seedlings in 5 days old. Date means ± SD (n = 3). The experiments were repeated three times with similar results.
(TIF)

S4 Fig The expression level of *UPB1* in transgenic seedlings. Expression analysis of *UPB1* in the roots of Col-0, *35S*::*UPB1-HA-Flag*, *35S*::*UPB1-HA-Flag/bin2-1*(+/-), and *35S*::*UPB1-HA-Flag/bin2-3bil1bil2* seedlings at 5 days old. Date means ± SD (n = 3). The experiments were repeated three times with similar results.
(TIF)

S5 Fig Subcellular localization of UPB1 and UPB1^S37AS41A^ in *N*. *benthamiana* leaves. GFP, UPB1-GFP, and UPB1^S37AS41A^-GFP were transformed into *N*. *benthamiana* leaves cells. Bar = 50 μm.
(TIF)

S6 Fig BIN2 increases UPB1 transcriptional activity. (A-C) Transient gene expression assays were performed in *upb1-1* protoplasts with the indicated gene promoters; LUC reporter genes were co-transfected with UPB1 and/or BIN2. The relative expression levels of LUC were normalized to those of REN. (D-F) BIN2 influences the DNA-binding activity of UPB1. ChIP-qPCR assays were performed using 14-day-old Col-0, *35S*::*UPB1-HA-Flag*, and *35S*::*UPB1-HA-Flag/bin2-1*(+/-) seedlings, treated with or without 50 μM MG132 before harvesting the samples. Chromatin fragments (~500 bp) were immunoprecipitated by anti-HA agarose beads (IP) or native agarose beads (Mock). The precipitated DNA was analyzed by qPCR using the primer pairs of *At4g11290* (D), *At4g16270* (E), *At5g17820* (F), and TA3 as negative controls. The level of binding was calculated as the ratio between IP and Mock and normalized to that of TA3 as an internal control. Double asterisk represent highly significant differences, (**, P<0.01; Student’s *t* test). The experiments were repeated three times with similar results.
(TIF)

S7 Fig UPB1 does not interact with the BES1 protein and influences its phosphorylation level. (A) BiFC assays. nYFP together with cYFP, BES1-nYFP together with cYFP, nYFP together with UPB1-cYFP, and BES1-nYFP together with UPB1-cYFP were co-transformed into *N*. *benthamiana* leaf cells. Bar = 50 μm. (B) Y2H assays of the interaction between BES1 and UPB1. Transformed yeast cells were grown on the SD-L-W or SD-L-W-H medium. (C) Ten-day-old Col-0, *upb1-1*, *35S*::*UPB1-HA-Flag#3*, and *35S*::*UPB1-HA-Flag#8* seedlings was treated without or with 1 μM BL. Samples were collected at 4 h time points. BES1 was detected with an anti-BES1 antibody. Actin was used as a control.
(TIF)

S8 Fig PRE2/3 interacts with UPB1 in BiFC assays. BiFC assays. PRE1-nYFP together with UPB1-cYFP, PRE2-nYFP together with UPB1-cYFP, PRE3-nYFP together with UPB1-cYFP, PRE4-nYFP together with UPB1-cYFP, PRE5-nYFP together with UPB1-cYFP, and PRE6-nYFP together with UPB1-cYFP were co-transformed into *N*. *benthamiana* leaf cells. DAPI staining was used as a nuclear marker. Bar = 50 μm.
(TIF)

S9 Fig Morphological features of PRE2/3 transgenic seedlings. (A) Phenotypes of 5-day-old seedlings of Col-0, *pre-amiR*, *pre3*, *pre-amiR/pre3*, *35S*::*PRE2-GFP*, and *35S*::*PRE3-GFP*. Bar = 0.5 cm. (B) Root meristem of Col-0, *pre-amiR*, *pre3*, *pre-amiR/pre3*, *35S*::*PRE2-GFP*, and *35S*::*PRE3-GFP* in 5-day-old seedlings. White arrowheads (below) mark the position of the quiescent center (QC), and white arrowheads (above) mark the end of the meristem where cells start to elongate. Bar = 50 μm. The primary root length (C), meristem size (D), meristem cell number (E), and meristem cell size (F) of the seedlings shown in (A). Date means ± SD (n≥20). Double asterisk represent highly significant differences (**, P<0.01; Student’s *t* test). (G) Expression analysis of *PRE1/2/3/4/5/6* in the roots of Col-0 and *pre-amiR/pre3* seedlings at 5 days old. The experiments were repeated three times with similar results. (H) Expression analysis of *PRE2/3* in the roots of Col-0, *35S*::*PRE2-GFP#3*, *35S*::*PRE2-GFP#5*, *35S*::*PRE3-GFP#1*, and *35S*::*PRE3-GFP#12* seedlings at 5 days old. The experiments were repeated three times with similar results.
(TIF)

S10 Fig The expression levels of *PRE2* and *PRE3* in BL response. Expression analysis of *PRE2*/*3* in the roots of Col-0; 5-day-old seedlings were treated with 100 nM BL for 3 h. The experiments were repeated three times with similar results.
(TIF)

S11 Fig Subcellular localization of PRE2/3 in *N*. *benthamiana* leaves and transgenic *Arabidopsis*. (A) PRE2-GFP and PRE3-GFP were transformed in *N*. *benthamiana* leaf cells. Bar = 50 μm. (B) Nuclear and total protein extracts from *35S*::*PRE2-GFP/35S*::*UPB1-HA-Flag*, *35S*::*PRE2-GFP*, *35S*::*PRE3-GFP/35S*::*UPB1-HA-Flag*, and *35S*::*PRE3-GFP* seedlings at 14 days old. PRE2/3 was detected with an anti-GFP antibody. Actin was used as a control. The bands were repeated twice.
(TIF)

S12 Fig PRE2/3 represses UPB1 transcriptional activity. (A-C) Transient gene expression assays were performed in *upb1-1* protoplasts with indicated the gene promoters-LUC reporter genes were co-transfected with UPB1 and/or PRE2, and UPB1 and/or PRE3. The relative expression levels of LUC were normalized to those of REN. (D-F) PRE2/3 influences the DNA-binding activity of UPB1. ChIP-qPCR assays were performed using 14-day-old Col-0, *35S*::*UPB1-HA-Flag*, *35S*::*PRE2-GFP/35S*::*UPB1-HA-Flag*, and *35S*::*PRE3-GFP/35S*::*UPB1-HA-Flag* seedlings. Chromatin fragments (~500 bp) were immunoprecipitated by HA agarose beads (IP) or native agarose beads (Mock). The precipitated DNA was analyzed by qPCR using the primer pairs of *At4g11290* (D), *At4g16270* (E), *At5g17820* (F), and TA3 as negative controls. The level of binding was calculated as the ratio between IP and Mock and normalized to that of TA3 as an internal control. Double asterisk represent highly significant differences, and asterisk represent significant differences, (**, P<0.01; *, P<0.05; Student’s *t* test). The experiments were repeated three times with similar results.
(TIF)

S13 Fig The expression levels of *PRE2/3* in Col-0 and *bin2-1*(+/+) seedlings. Expression analysis of *PRE2/3* in the roots of Col-0 and *bin2-1*(+/+) seedlings at 5 days old. Date means ± SD (n = 3). The experiments were repeated three times with similar results.
(TIF)

S1 Data Underlying data for Figs 1C, 1D, 1E, 3A, 3B, 3C, 3D, 3G, 3H, 3I, 4D, 4E, 4F, 5D, 5F, 5G, 5H, 6C, 7E, 7F 7G, 8D, 8E and 8F. (XLSX)

S2 Data S2D, S2E, S2F, S2G, S3B, S3C, S4, S6A, S6B, S6C, S6D, S6E, S6F, S9C, S9D, S9E, S9F, S9G, S9H, S10, S12A, S12B, S12C, S12D, S12E, S12F, S13. (XLSX)

S1 Table Primer sequences used in this study. (DOCX)
