# Supplementary figures and images for: Brassinosteroids regulate root meristem development by mediating BIN2-UPB1 module in Arabidopsis
Source: PLoS Genet. 2020 Jul 1;16(7):e1008883. doi: 10.1371/journal.pgen.1008883 (PMC7360063; doi:10.1371/journal.pgen.1008883)

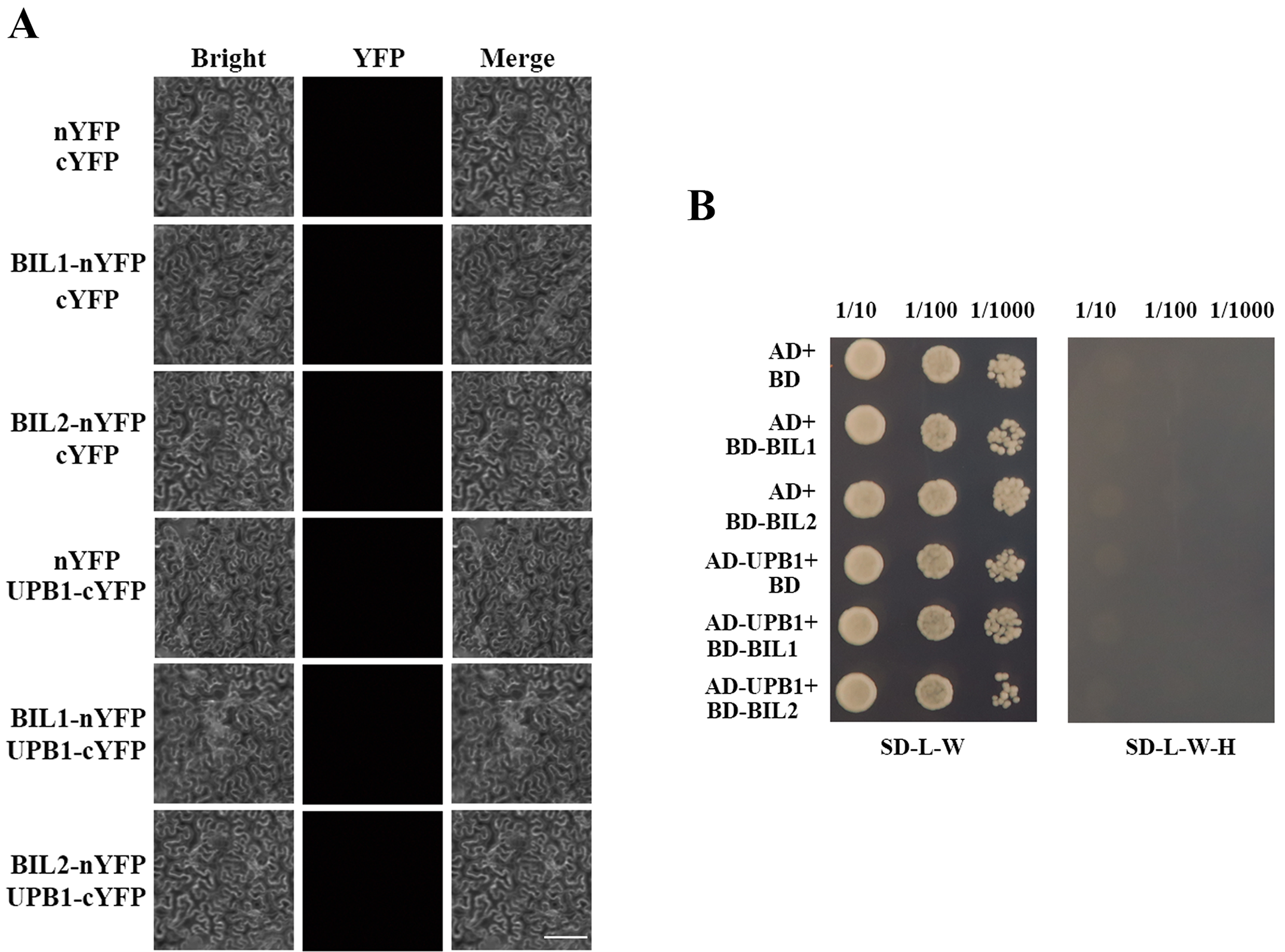

Supplement: S1 Fig — (A) In BiFC assays. nYFP and cYFP, BIL1-nYFP and cYFP, BIL2-nYFP and cYFP, nYFP and UPB1-cYFP, BIL1-nYFP and UPB1-cYFP, and BIL2-nYFP and UPB1-cYFP were co-transformed in N. benthamiana leaves cells. Bar = 50 μm. (B) Y2H assays of the interaction between BIL1/2 and UPB1. Transformed yeast cells were grown on the SD-L-W or SD-L-W-H medium. (TIF) [file pgen.1008883.s001.tif]

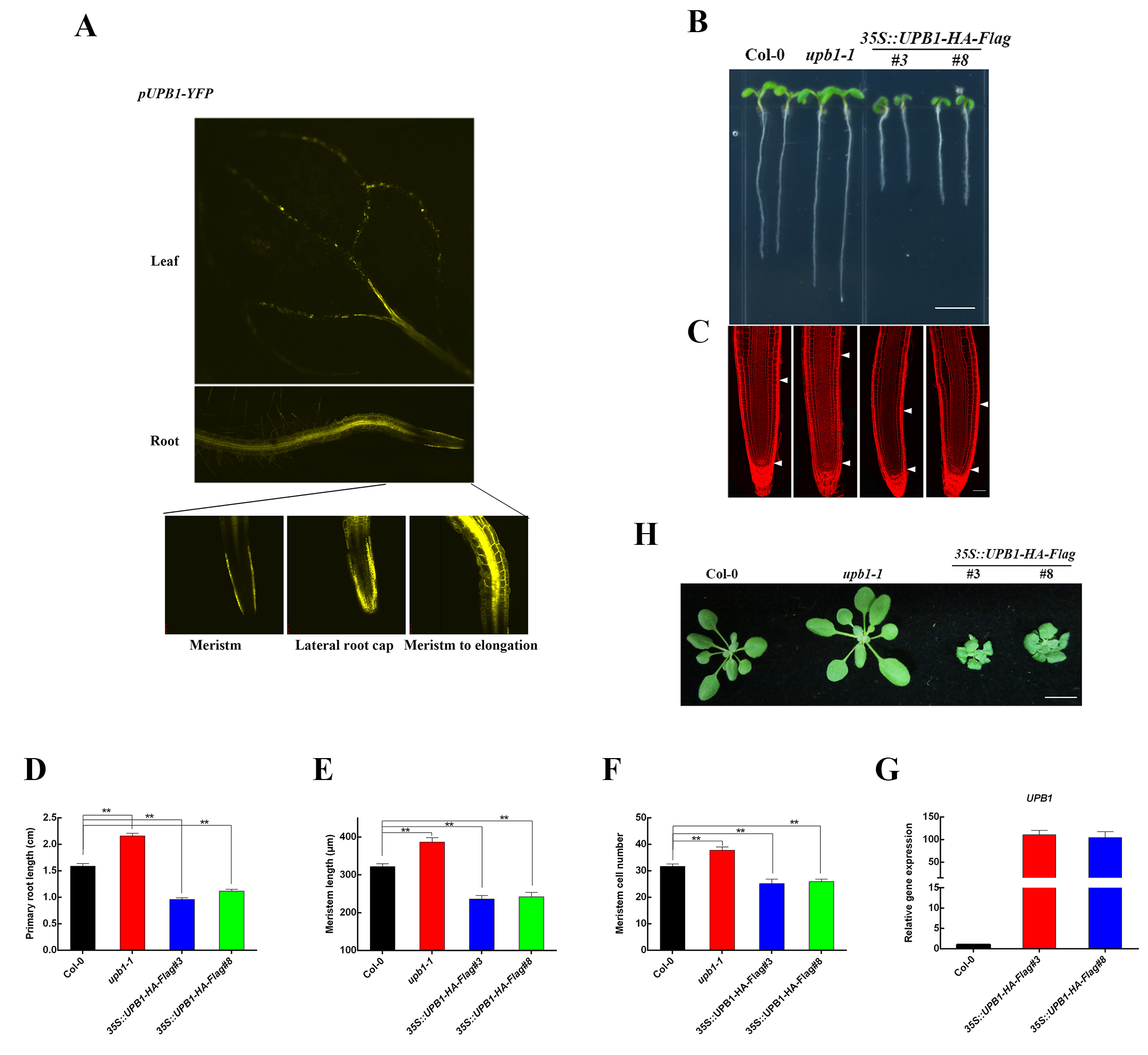

Supplement: S2 Fig — (A) The expression profiles of UPB1 in Arabidopsis. Bar = 50 μm. (B) Phenotypes of 5-day-old seedlings of Col-0, upb1-1, 35S::UPB1-HA-Flag#3, and 35S::UPB1-HA-Flag#8. Bar = 0.5 cm. (C) Root meristem of Col-0, upb1-1, 35S::UPB1-HA-Flag#3, and 35S::UPB1-HA-Flag#8 in 5-day-old seedlings. White arrowheads (below) mark the position of the quiescent center (QC), and white arrowheads (above) mark the end of the meristem where cells start to elongate. Bar = 50 μm. The primary root length (D), meristem size (E), and meristem cell number (F) of the seedlings shown in (B). Date means ± SD (n≥20). Double asterisk represent highly significant differences (**, P<0.01; Student’s t test). (G) Expression analysis of UPB1 in the roots of Col-0, 35S::UPB1-HA-Flag#3, and 35S::UPB1-HA-Flag#8 seedlings at 5 days old. (H) Phenotypes of 4-week-old seedlings of Col-0, upb1-1, 35S::UPB1-HA-Flag#3, and 35S::UPB1-HA-Flag#8. Bar = 1 cm. (TIF) [file pgen.1008883.s002.tif]

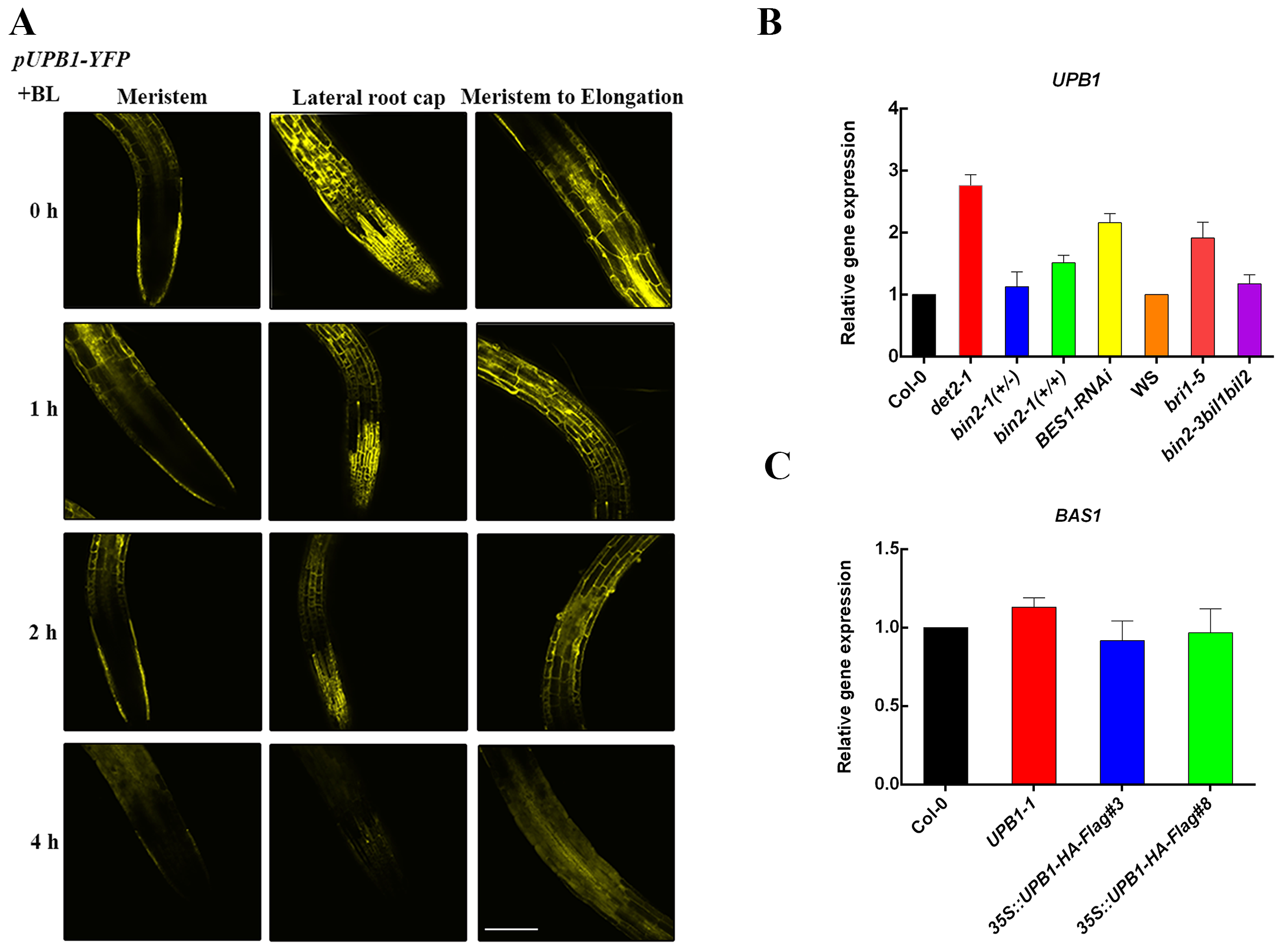

Supplement: S3 Fig — (A) pUPB1-YFP transgenic seedlings was absence or presence of 100 nM BL, and the fluorescence signal was detected at 0 h, 1 h, 2 h, and 4 h. Bar = 50 μm. (B) The expression of UPB1 was examined by RT-qPCR in the roots of Col-0, det2-1, bin2-1(+/-), bin2-1(+/+), BES1-RNAi, WS, bri1-5, and bin2-3bil1bil2 seedlings at 5 days old. (C) Expression analysis of BAS1 in the roots of Col-0, upb1-1, 35S::UPB1-HA-Flag#3, and 35S::UPB1-HA-Flag#8 seedlings in 5 days old. Date means ± SD (n = 3). The experiments were repeated three times with similar results. (TIF) [file pgen.1008883.s003.tif]

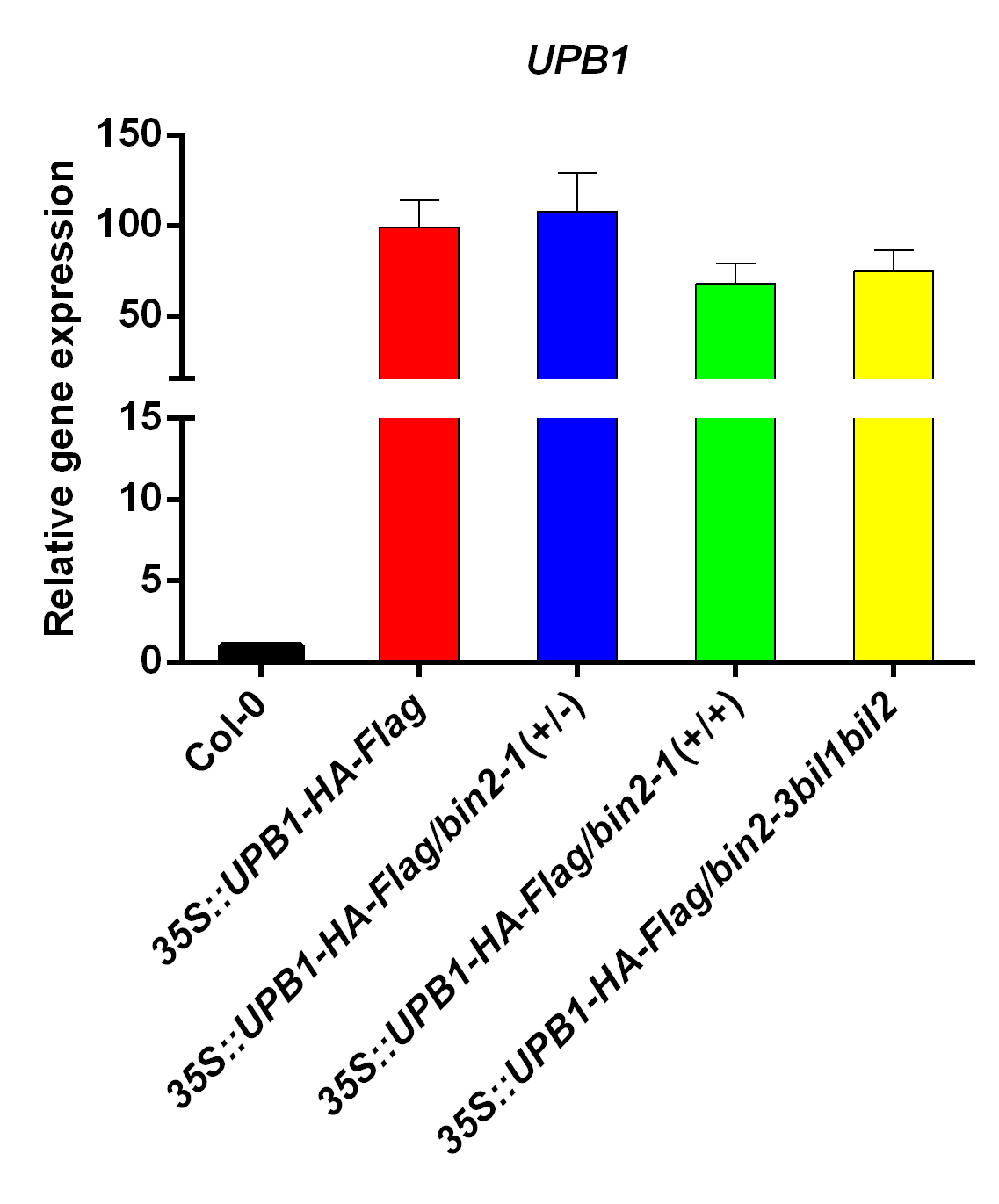

Supplement: S4 Fig — Expression analysis of UPB1 in the roots of Col-0, 35S::UPB1-HA-Flag, 35S::UPB1-HA-Flag/bin2-1(+/-), and 35S::UPB1-HA-Flag/bin2-3bil1bil2 seedlings at 5 days old. Date means ± SD (n = 3). The experiments were repeated three times with similar results. (TIF) [file pgen.1008883.s004.tif]

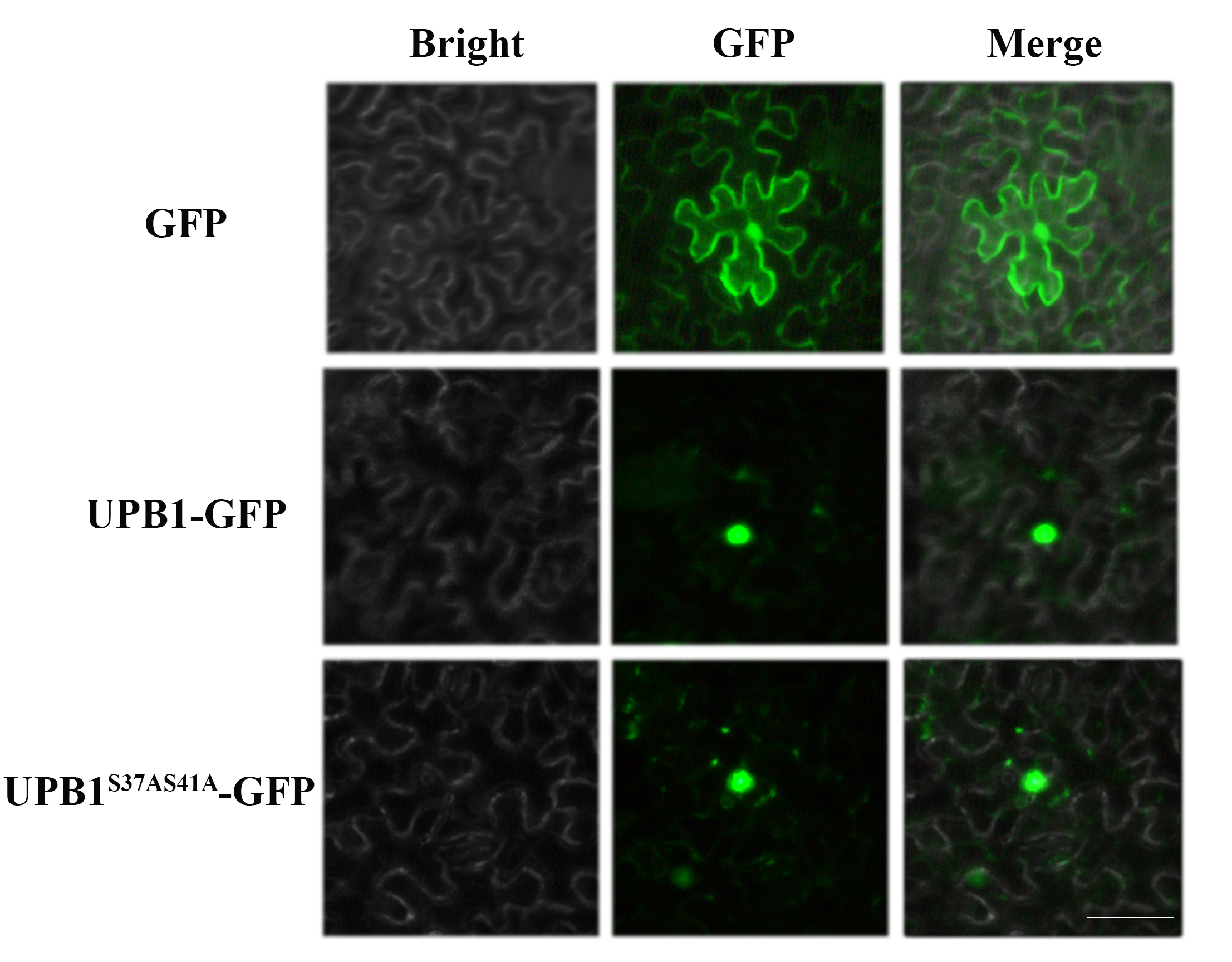

Supplement: S5 Fig — GFP, UPB1-GFP, and UPB1S37AS41A-GFP were transformed into N. benthamiana leaves cells. Bar = 50 μm. (TIF) [file pgen.1008883.s005.tif]

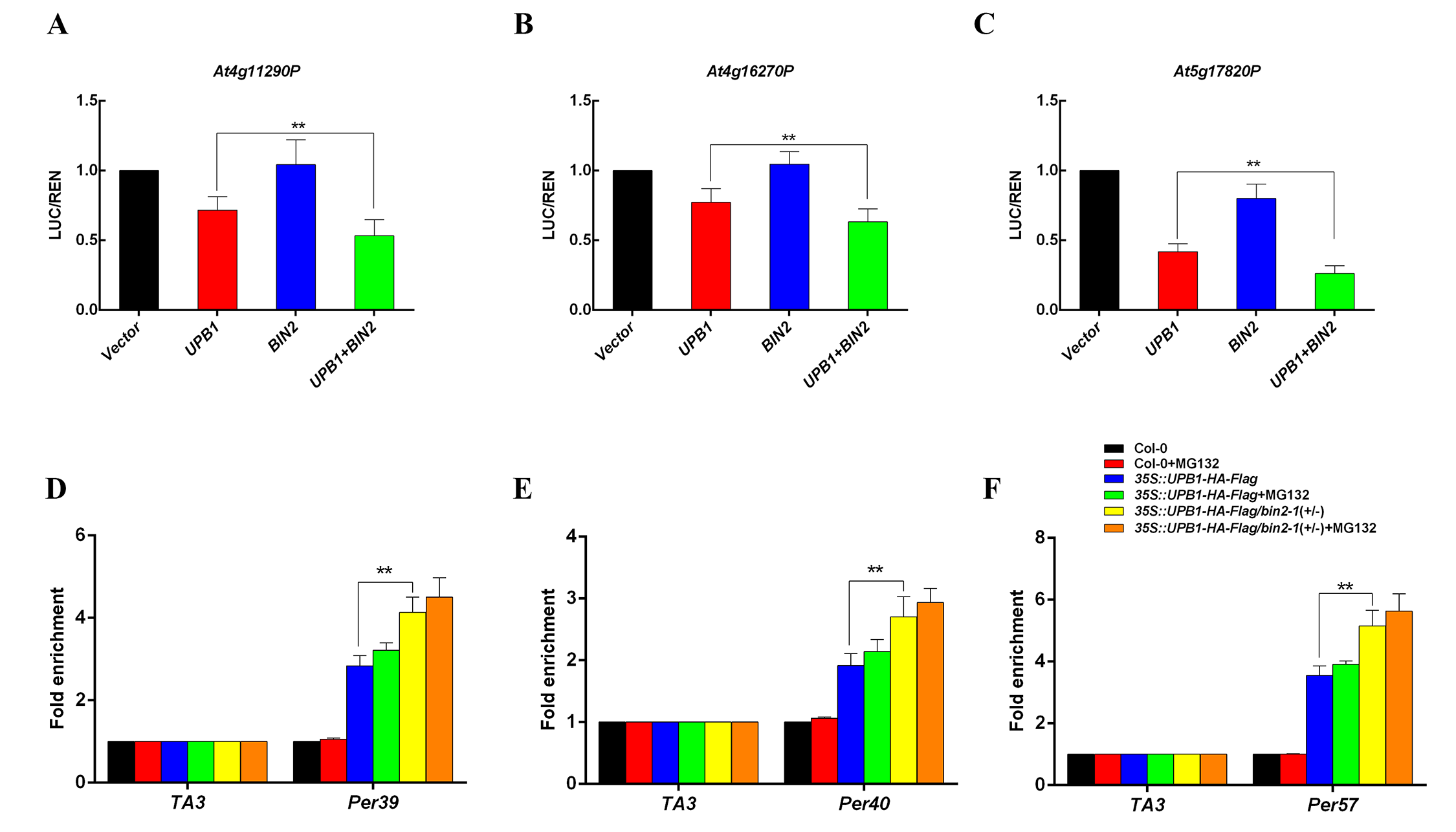

Supplement: S6 Fig — (A-C) Transient gene expression assays were performed in upb1-1 protoplasts with the indicated gene promoters; LUC reporter genes were co-transfected with UPB1 and/or BIN2. The relative expression levels of LUC were normalized to those of REN. (D-F) BIN2 influences the DNA-binding activity of UPB1. ChIP-qPCR assays were performed using 14-day-old Col-0, 35S::UPB1-HA-Flag, and 35S::UPB1-HA-Flag/bin2-1(+/-) seedlings, treated with or without 50 μM MG132 before harvesting the samples. Chromatin fragments (~500 bp) were immunoprecipitated by anti-HA agarose beads (IP) or native agarose beads (Mock). The precipitated DNA was analyzed by qPCR using the primer pairs of At4g11290 (D), At4g16270 (E), At5g17820 (F), and TA3 as negative controls. The level of binding was calculated as the ratio between IP and Mock and normalized to that of TA3 as an internal control. Double asterisk represent highly significant differences, (**, P<0.01; Student’s t test). The experiments were repeated three times with similar results. (TIF) [file pgen.1008883.s006.tif]

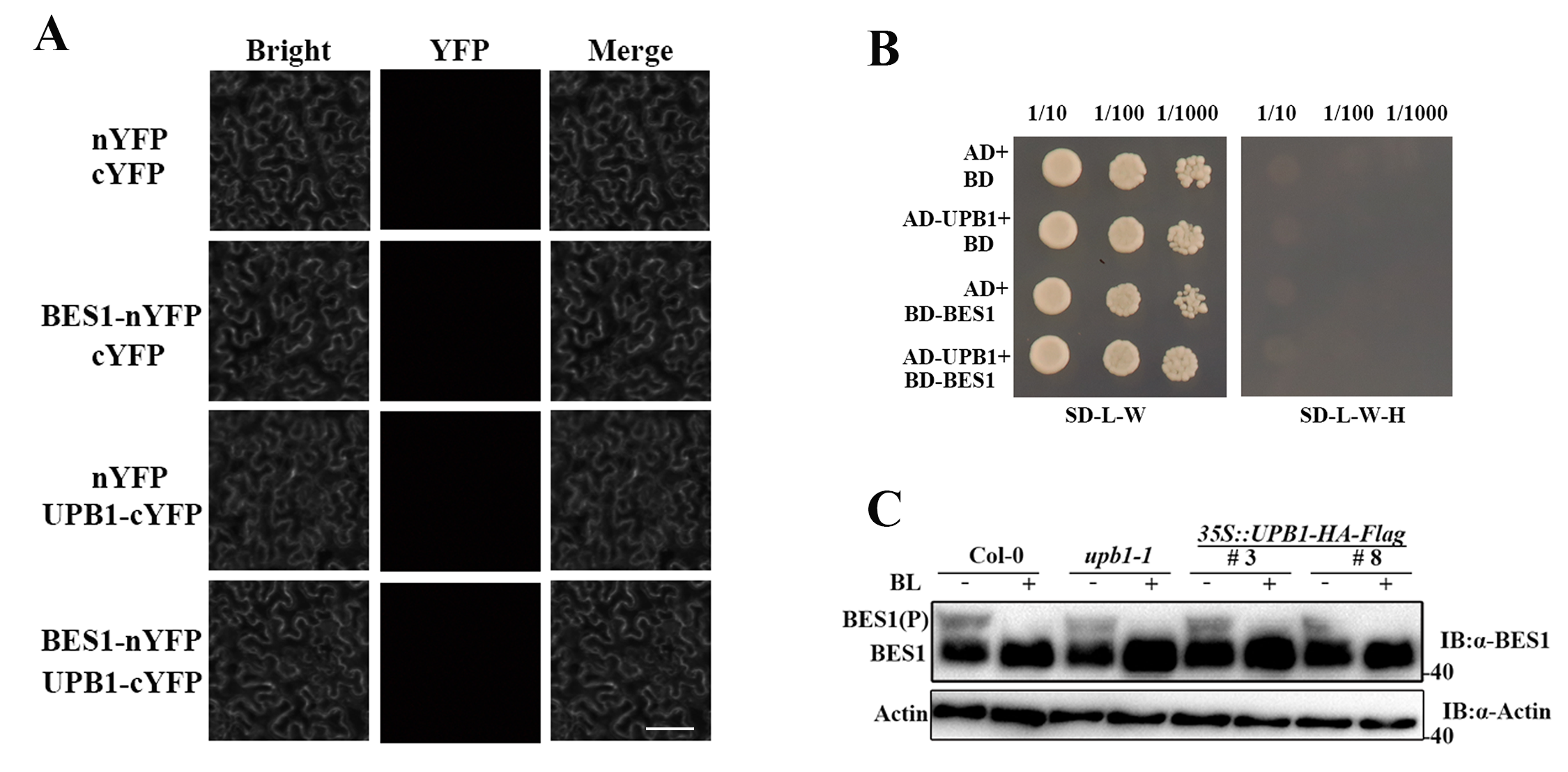

Supplement: S7 Fig — (A) BiFC assays. nYFP together with cYFP, BES1-nYFP together with cYFP, nYFP together with UPB1-cYFP, and BES1-nYFP together with UPB1-cYFP were co-transformed into N. benthamiana leaf cells. Bar = 50 μm. (B) Y2H assays of the interaction between BES1 and UPB1. Transformed yeast cells were grown on the SD-L-W or SD-L-W-H medium. (C) Ten-day-old Col-0, upb1-1, 35S::UPB1-HA-Flag#3, and 35S::UPB1-HA-Flag#8 seedlings was treated without or with 1 μM BL. Samples were collected at 4 h time points. BES1 was detected with an anti-BES1 antibody. Actin was used as a control. (TIF) [file pgen.1008883.s007.tif]

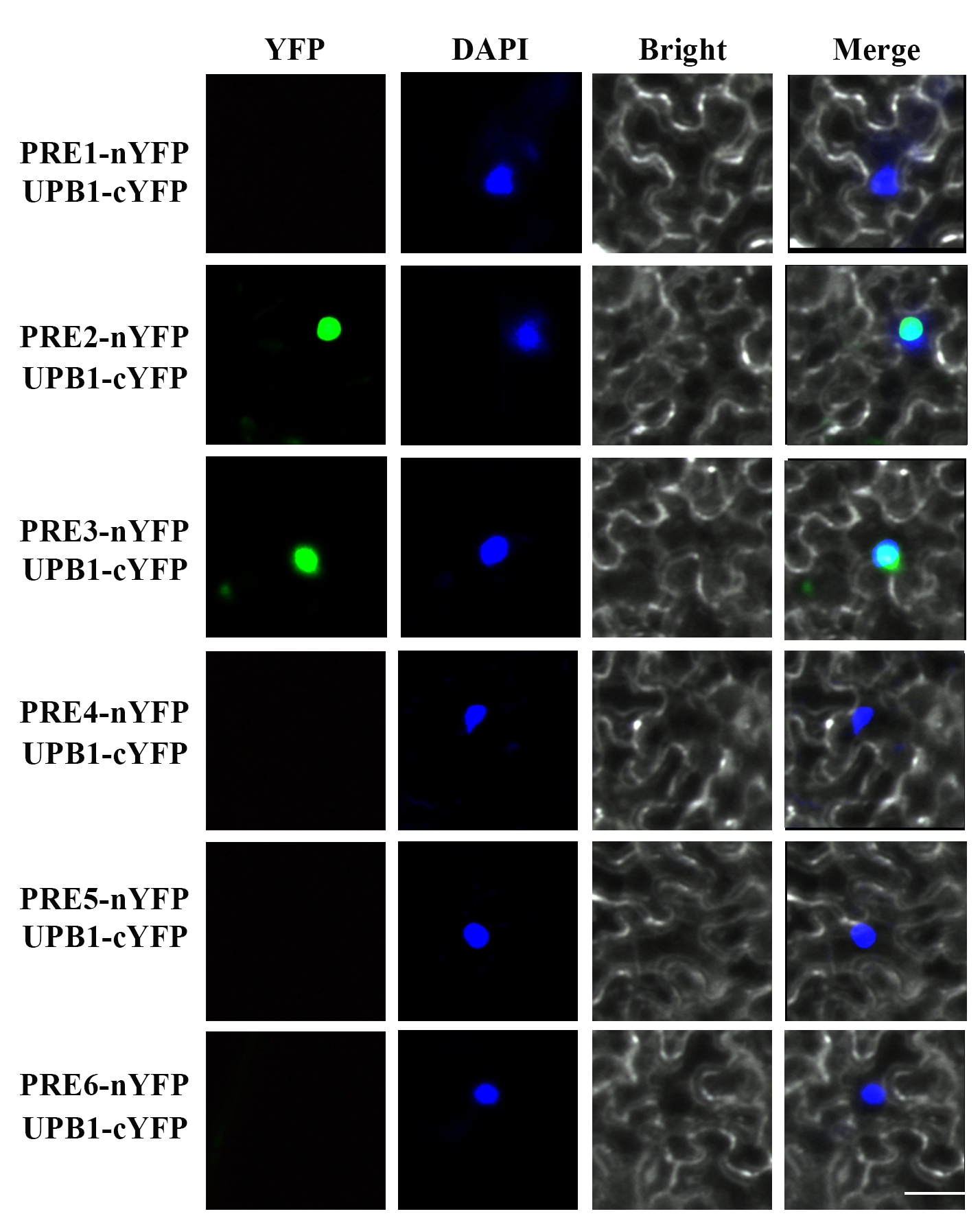

Supplement: S8 Fig — BiFC assays. PRE1-nYFP together with UPB1-cYFP, PRE2-nYFP together with UPB1-cYFP, PRE3-nYFP together with UPB1-cYFP, PRE4-nYFP together with UPB1-cYFP, PRE5-nYFP together with UPB1-cYFP, and PRE6-nYFP together with UPB1-cYFP were co-transformed into N. benthamiana leaf cells. DAPI staining was used as a nuclear marker. Bar = 50 μm. (TIF) [file pgen.1008883.s008.tif]

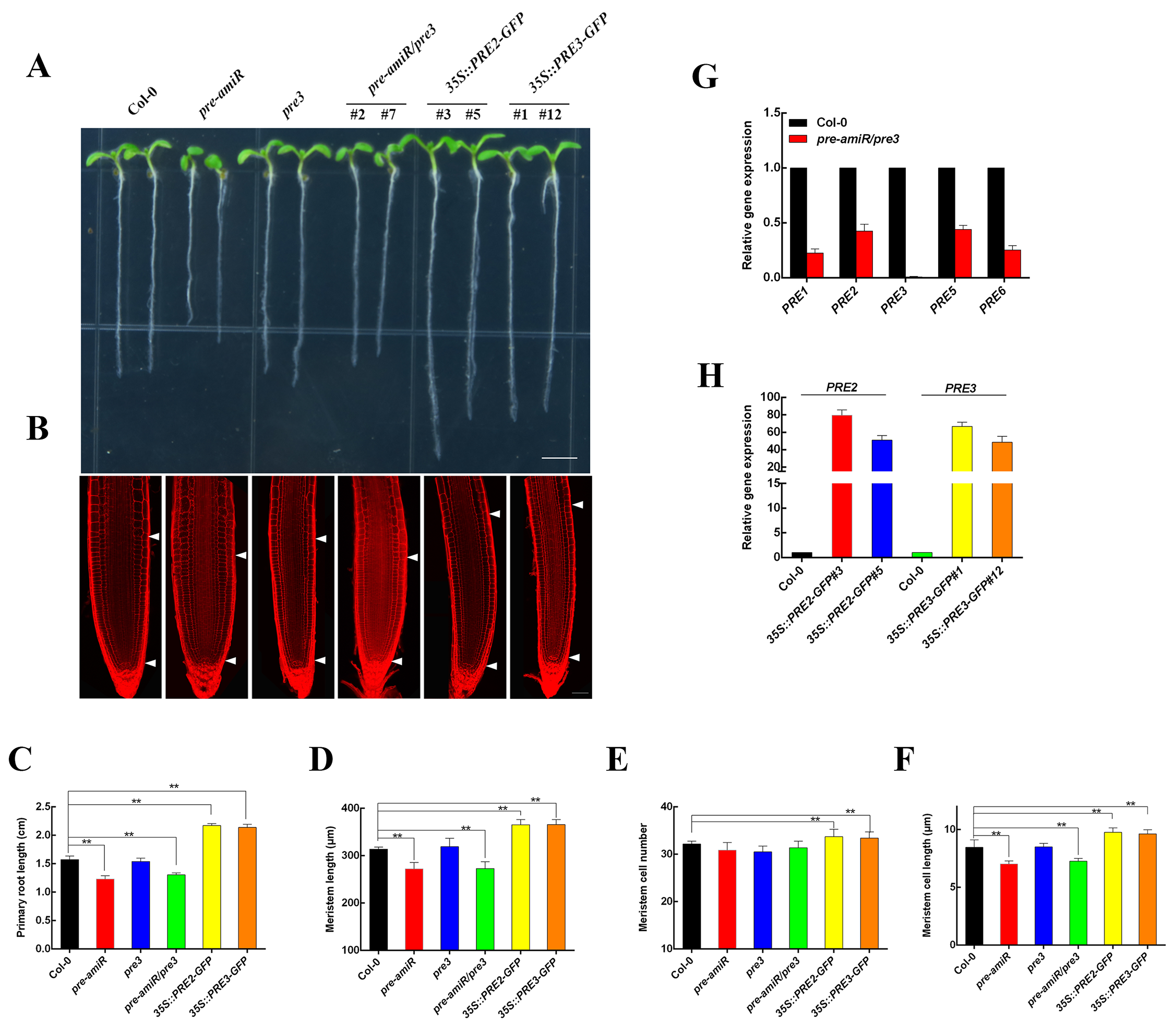

Supplement: S9 Fig — (A) Phenotypes of 5-day-old seedlings of Col-0, pre-amiR, pre3, pre-amiR/pre3, 35S::PRE2-GFP, and 35S::PRE3-GFP. Bar = 0.5 cm. (B) Root meristem of Col-0, pre-amiR, pre3, pre-amiR/pre3, 35S::PRE2-GFP, and 35S::PRE3-GFP in 5-day-old seedlings. White arrowheads (below) mark the position of the quiescent center (QC), and white arrowheads (above) mark the end of the meristem where cells start to elongate. Bar = 50 μm. The primary root length (C), meristem size (D), meristem cell number (E), and meristem cell size (F) of the seedlings shown in (A). Date means ± SD (n≥20). Double asterisk represent highly significant differences (**, P<0.01; Student’s t test). (G) Expression analysis of PRE1/2/3/4/5/6 in the roots of Col-0 and pre-amiR/pre3 seedlings at 5 days old. The experiments were repeated three times with similar results. (H) Expression analysis of PRE2/3 in the roots of Col-0, 35S::PRE2-GFP#3, 35S::PRE2-GFP#5, 35S::PRE3-GFP#1, and 35S::PRE3-GFP#12 seedlings at 5 days old. The experiments were repeated three times with similar results. (TIF) [file pgen.1008883.s009.tif]

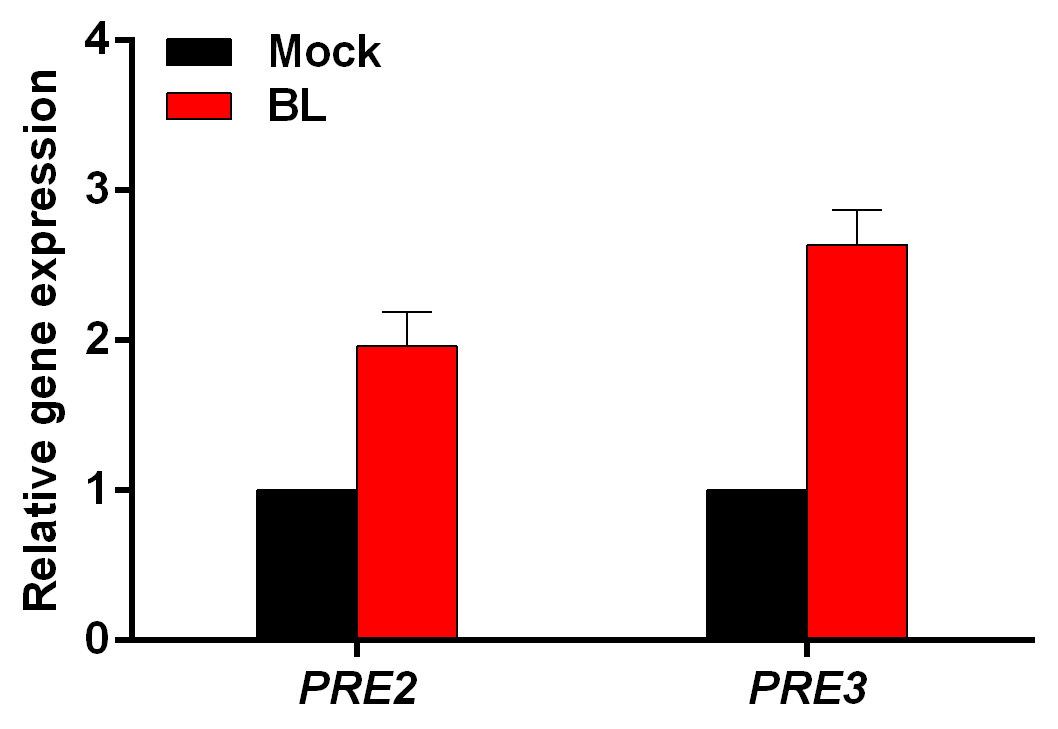

Supplement: S10 Fig — Expression analysis of PRE2/3 in the roots of Col-0; 5-day-old seedlings were treated with 100 nM BL for 3 h. The experiments were repeated three times with similar results. (TIF) [file pgen.1008883.s010.tif]

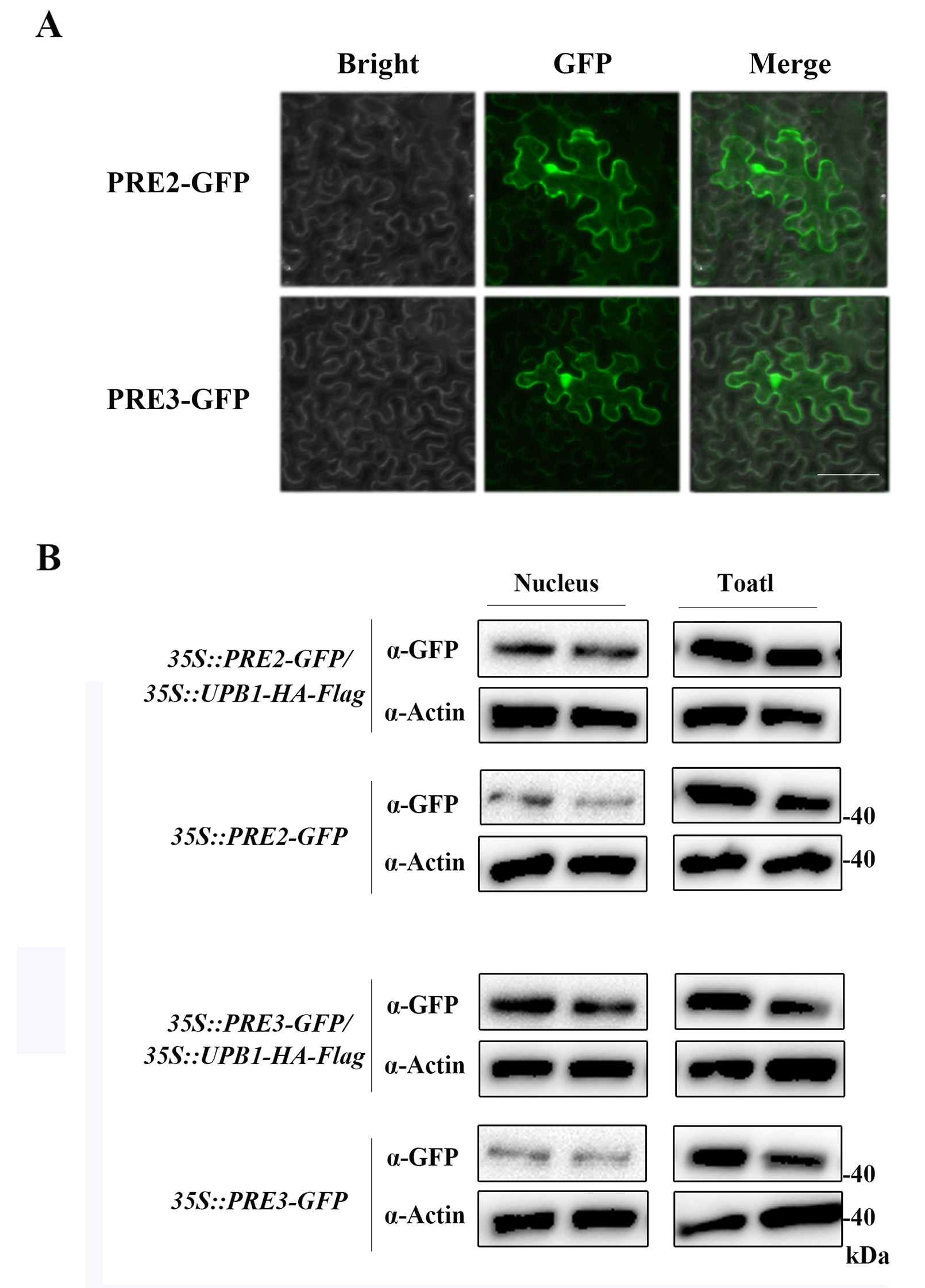

Supplement: S11 Fig — (A) PRE2-GFP and PRE3-GFP were transformed in N. benthamiana leaf cells. Bar = 50 μm. (B) Nuclear and total protein extracts from 35S::PRE2-GFP/35S::UPB1-HA-Flag, 35S::PRE2-GFP, 35S::PRE3-GFP/35S::UPB1-HA-Flag, and 35S::PRE3-GFP seedlings at 14 days old. PRE2/3 was detected with an anti-GFP antibody. Actin was used as a control. The bands were repeated twice. (TIF) [file pgen.1008883.s011.tif]

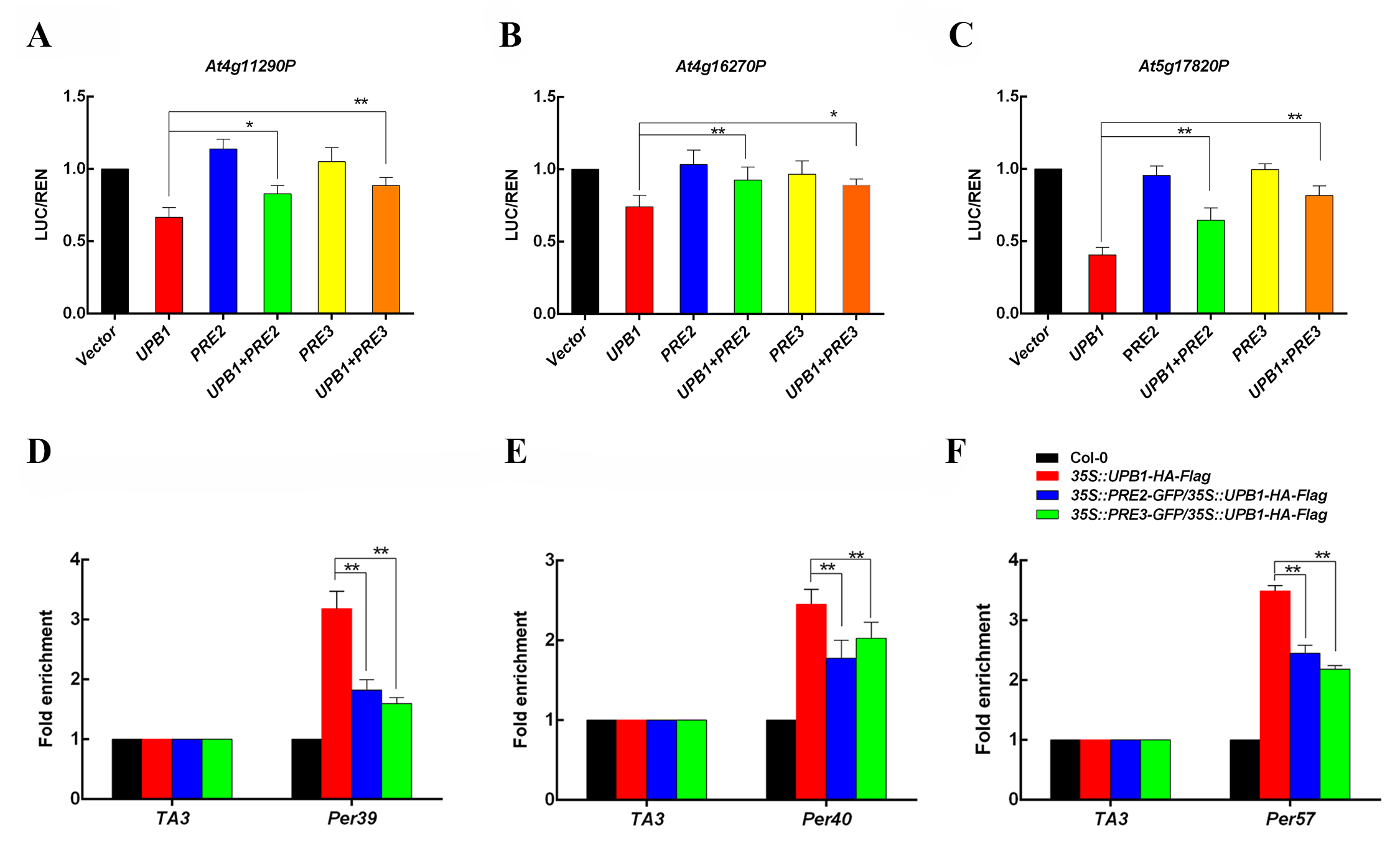

Supplement: S12 Fig — (A-C) Transient gene expression assays were performed in upb1-1 protoplasts with indicated the gene promoters-LUC reporter genes were co-transfected with UPB1 and/or PRE2, and UPB1 and/or PRE3. The relative expression levels of LUC were normalized to those of REN. (D-F) PRE2/3 influences the DNA-binding activity of UPB1. ChIP-qPCR assays were performed using 14-day-old Col-0, 35S::UPB1-HA-Flag, 35S::PRE2-GFP/35S::UPB1-HA-Flag, and 35S::PRE3-GFP/35S::UPB1-HA-Flag seedlings. Chromatin fragments (~500 bp) were immunoprecipitated by HA agarose beads (IP) or native agarose beads (Mock). The precipitated DNA was analyzed by qPCR using the primer pairs of At4g11290 (D), At4g16270 (E), At5g17820 (F), and TA3 as negative controls. The level of binding was calculated as the ratio between IP and Mock and normalized to that of TA3 as an internal control. Double asterisk represent highly significant differences, and asterisk represent significant differences, (**, P<0.01; *, P<0.05; Student’s t test). The experiments were repeated three times with similar results. (TIF) [file pgen.1008883.s012.tif]

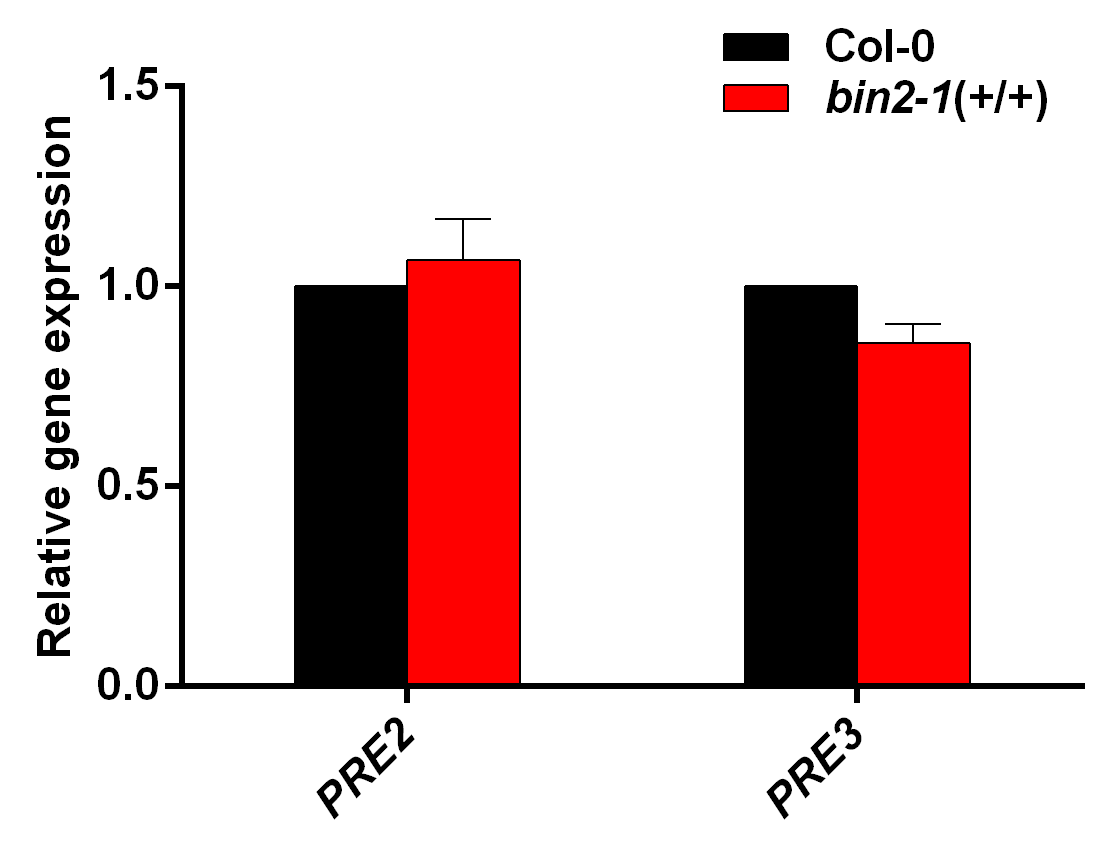

Supplement: S13 Fig — Expression analysis of PRE2/3 in the roots of Col-0 and bin2-1(+/+) seedlings at 5 days old. Date means ± SD (n = 3). The experiments were repeated three times with similar results. (TIF) [file pgen.1008883.s013.tif]
