# Supplementary material for: Brassinosteroids regulate root meristem development by mediating BIN2-UPB1 module in Arabidopsis
Source: PLoS Genet. 2020 Jul 1;16(7):e1008883. doi: 10.1371/journal.pgen.1008883 (PMC7360063; doi:10.1371/journal.pgen.1008883)
Supplement: S1 Table — (DOCX) [file pgen.1008883.s016.docx]

**Supplemental Table 1**

| Primer name | Sequences |
| --- | --- |
| UPB1-HA-Flag-F1 | 5’-ACGCGTCGACATGGGTGTAACATTAGAAGGAC-3’ |
| UPB1-HA-Flag-R1 | 5’-CGGGGTACCAACACAGTTAGTTTCGGTCAAA-3’ |
| UPB1^S37AS41A^-HA-Flag-F1 | 5’-TCCGACCAAGGAAGGCTGTAGAAGCTGCTAGAAGACCTTGTC-3’ |
| UPB1^S37AS41A^-HA-Flag-R1 | 5’-GACAAGGTCTTCTAGCAGCTTCTACAGCCTTCCTTGGTCGGA-3’ |
| pUPB1-HA-Flag-F1 | 5’-CCGGAATTCATGGGTGTAACATTAGAAGGAC-3’ |
| pUPB1-HA-Flag-R1 | 5’-CGCGGATCCAACACAGTTAGTTTCGGTCAAA-3’ |
| UPB1-Yeast-F1 | 5’-CCGGAATTCATGGGTGTAACATTAGAAGGAC-3’ |
| UPB1-Yeast-R1  BIN2-Yeast-F1  BIN2-Yeast-R1  BIL1-Yeast-F1  BIL1-Yeast-R1  BIL2-Yeast-F1  BIL2-Yeast-R1  BES1-Yeast-F1  BES1-Yeast-R1 | 5’-GCGAGCTCTTAAACACAGTTAGTTTCGGTCA-3’  5’-CGCGGATCCATGGCTGATGATAAGGAGAT -3’  5’- ACGCGTCGACAGTTCCAGATTGATTCAAG-3’  5’-CCGGAATTCATGACTTCGATACCATTGGG -3’  5’-CGCGGATCCCTAGGGTCCAGCTTGAAATGG -3’  5’- CCGGAATTCATGGCCTCATTACCATTGG-3’  5’- CGCGGATCCTTAACTGTTTTGTAATCCTG-3’  5’-CCGGAATTCATGAAAAGATTCTTCTATAATTCCA-3’  5’-GCCGAGCTCTCAACTATGAGCTTTACCATTT-3’ |
| UPB1-EMSA-E5/E6-F1 | 5’-AGTGATGATAATCATGTGAGTGCATGTGCATCGAGTTGTA-3’ |
| UPB1-EMSA-E5/E6-R1 | 5’-TACAACTCGATGCACATGCACTCACATGATTATCATCACT-3’ |
| UPB1-EMSA-mE5/E6-F1 | 5’-AGTGATGATAATTCTGGAAGTGCATGTGCATCGAGTTGTA-3’ |
| UPB1-EMSA-mE5/E6-R1 | 5’-TACAACTCGATGCACATGCACTTCCAGAATTATCATCACT-3’ |
| UPB1-EMSA-E5/mE6-F1 | 5’-AGTGATGATAATCATGTGAGTGTCTGGACATCGAGTTGTA-3’ |
| UPB1-EMSA-E5/mE6-R1 | 5’-TACAACTCGATGTCCAGACACTCACATGATTATCATCACT-3’ |
| UPB1-EMSA-mE5/mE6-F1 | 5’-AGTGATGATAATTCTGGAAGTGTCTGGACATCGAGTTGTA-3’ |
| UPB1-EMSA-mE5/mE6-R1 | 5’-TACAACTCGATGTCCAGACACTTCCAGAATTATCATCACT -3’ |
| UPB1-EMSA-E7-F1 | 5’-AAACACCCTTGTGGACACTTGTCAAATTCTCATCCA -3’ |
| UPB1-EMSA-E7-R1 | 5’-TGGATGAGAATTTGACAAGTGTCCACAAGGGTGTTT -3’ |
| UPB1-EMSA-E8-F1 | 5’-GTAAGCAAACGTTATCACTTGTCTACACAACATTCT -3’ |
| UPB1-EMSA-E8-R1 | 5’-AGAATGTTGTGTAGACAAGTGATAACGTTTGCTTAC -3’ |
| UPB1-His-SUMO-F1 | 5’-CGCGGATCCATGGGTGTAACATTAGAAGGAC -3’ |
| UPB1-His-SUMO-R1 | 5’-ACGCGTCGACTTAAACACAGTTAGTTTCGGTCA -3’ |
| BIN2-MBP-F1 | 5’-CGCGGATCCATGGCTGATGATAAGGAGAT-3’ |
| BIN2-MBP-R1 | 5’-ACGCGTCGACAGTTCCAGATTGATTCAAG-3’ |
| BES1-MBP-F1 | 5’-ATACCCGGGATGAAAAGATTCTTCTATAA-3’ |
| BES1-MBP-R1 | 5’-CGCGGATCCACTATGAGCTTTACCAT-3’ |
| PRE2-pGEX-4T-F1 | 5’-CGCGGATCCATGTCTTCTAGCAGAAGGTCGA-3’ |
| PRE2-pGEX-4T-R1 | 5’-ACGCGTCGACTCCATTAATCAAGCTCCTAATAAC-3’ |
| PRE3-pGEX-4T-F1 | 5’-CGCGGATCCATGTCGGGAAGAAGATCACG-3’ |
| PRE3-pGEX-4T-R1 | 5’-ACGCGTCGACTTGGGTAAGTAAGCTTCTGATTA-3’ |
| PRE2-GFP-F1 | 5’-CGGGGTACCATGTCTTCTAGCAGAAGGTCGA-3’ |
| PRE2-GFP-R1 | 5’-CGCGGATCCTCCATTAATCAAGCTCCTAATAAC -3’ |
| PRE3-GFP-F1 | 5’-CGGGGTACCATGTCGGGAAGAAGATCACG-3’ |
| PRE3-GFP-R1 | 5’-CGCGGATCCTTGGGTAAGTAAGCTTCT-3’ |
| pUPB1-Y1H-F | 5’-CCGGAATTCCGGCAATCAATTAAAACAAACAT-3’ |
| pUPB1-Y1H-R | 5’-CGACGCGTTCTTCACTCGTCTGTGTATTGCG-3’ |
| qRT-Actin-F1 | 5’- GGTAACATTGTGCTCAGTGGTGG-3’ |
| qRT-Actin-R1 | 5’-AACGACCTTAATCTTCATGCTGC-3’ |
| qRT-UPB1-F1 | 5’-ATGGGTGTAACATTAGAAGGAC-3’ |
| qRT-UPB1-R1 | 5’-TTCATTTCCAAAGCCAAGATA-3’ |
| qRT-PRE1-F1 | 5’-GTTCTGATAAGGCATCAGCCTCG -3’ |
| qRT-PRE1-R1 | 5’-CATGAGTAGGCTTCTAATAACGG -3’ |
| qRT-PRE2-F1 | 5’-CCGTCGTTCCAACACGGTATCA-3’ |
| qRT-PRE2-R1 | 5’-CTGCGGCTTGTGGGCTATTAGG-3’ |
| qRT-PRE3-F1 | 5’-ATCAGGAACTTCAAGGAT-3’ |
| qRT-PRE3-R1 | 5’-GCTAGTAACTCAGATAGCCT-3’ |
| qRT-PRE5-F1 | 5’-AACGGCGTCGTTCTGATAAG-3’ |
| qRT-PRE5-R1 | 5’-CATGAGTAAGCTTCTAATCACGG-3’ |
| qRT-PRE6-F1 | 5’-TCCAACACCTCATCCCTGAACTTCG-3’ |
| qRT-PRE6-R1  qRT-BAS1-F1  qRT-BAS1-R1 | 5’-CGGTCACTGAGGTCATCAACCTCTC-3’  5’-GCTCTCCTTTTTGTGTTTTCTCTCT-3’  5’-AGTCCGGAACAAATTTTTGACCGTT3’ |
| ChIP-qPCR-P1-F1 | 5’-TGAGCAAACCCAATTATATGTG-3’ |
| ChIP-qPCR-P1-R1 | 5’-CTAGGATTTAATCCGTGGTACAC-3’ |
| ChIP-qPCR-P2-F1 | 5’-TTGTGAATTGGGATATAGATGTG-3’ |
| ChIP-qPCR-P2-R1 | 5’-TGTACGTCTCTGATAATTGAGATAT-3’ |
| ChIP-qPCR-P3-F1 | 5’-ATTCAAAGGAACACAGAAGATCA-3’ |
| ChIP-qPCR-P3-R1 | 5’-GTGTTTGAGTTTCGCTACAATG-3’ |
| ChIP-qPCR-P4-F1 | 5’-CAAAGGAGGAATAAGACAGG-3’ |
| ChIP-qPCR-P4-R1 | 5’-ATTCCTTTCTTTGTCCTTCTA-3’ |
| ChIP-qPCR-Per39-F1 | 5’-CCAACAAAAAATAATGTCATAGAAC-3’ |
| ChIP-qPCR-Per39-R1 | 5’-CGTACGATGGCTATTTATTAGC-3’ |
| ChIP-qPCR-Per40-F1 | 5’-AATACAAATAAAGAAATACGAAGAG-3’ |
| ChIP-qPCR-Per40-R1 | 5’-TAAAAAGGAACAAAAGAGTAGTAAT-3’ |
| ChIP-qPCR-Per57-F1 | 5’-ATTTTTTACTGTTTGTGTTAGGTTA-3’ |
| ChIP-qPCR-Per57-R1 | 5’- GGATGAATTGAAAGTAGTGCTAA-3’ |
| TA3-F1 | 5’-GATTCTTACTGTAAAGAACATGGCATTGAGAGA-3’ |
| TA3-R1 | 5’-TCCAAATTTCCTGAGGTGCTTGTAACC-3’ |
| UPB1-BiFC-F1 | 5’-CGCGGATCCATGGGTGTAACATTAGAAGGAC-3’ |
| UPB1-BiFC-R1 | 5’-ACGCGTCGACAACACAGTTAGTTTCGGTCAAA-3’ |
| BES1-BiFC-F1 | 5’-ATACCCGGGATGAAAAGATTCTTCTATAA-3’ |
| BES1-BiFC-R1 | 5’-CGCGGATCCACTATGAGCTTTACCAT-3’ |
| BIN2-BiFC-F1 | 5’-CGCGGATCCATGGCTGATGATAAGGAGAT-3’ |
| BIN2-BiFC-R1 | 5’-ACGCGTCGACAGTTCCAGATTGATTCAAG-3’ |
| BIL1-BiFC-F1 | 5’-CGCGGATCCATGACTTCGATACCATTGGG-3’ |
| BIL1-BiFC-R1 | 5’-ACGCGTCGACGGGTCCAGCTTGAAATGG-3’ |
| BIL2-BiFC-F1 | 5’-CGCGGATCCATGGCCTCATTACCATTGG-3’ |
| BIL2-BiFC-R1 | 5’-ACGCGTCGACACTGTTTTGTAATCCTGTGCTC-3’ |
| PRE1-BiFC-F1 | 5’-CGCGGATCCATGTCGAACAGAAGATCAAGG-3’ |
| PRE1-BiFC-R1 | 5’-ACGCGTCGACCATGAGTAGGCTTCTAATAACGG-3’ |
| PRE2-BiFC-F1 | 5’-CGCGGATCCATGTCTTCTAGCAGAAGGTCGA-3’ |
| PRE2-BiFC-R1 | 5’-ACGCGTCGACTCCATTAATCAAGCTCCTAATAAC-3’ |
| PRE3-BiFC-F1 | 5’-CGCGGATCCATGTCGGGAAGAAGATCACG-3’ |
| PRE3-BiFC-R1 | 5’-ACGCGTCGACTTGGGTAAGTAAGCTTCTGATTA-3’ |
| PRE4-BiFC-F1 | 5’-CGCGGATCCATGTCTAGCAGAAAATCACGTTC-3’ |
| PRE4-BiFC-R1 | 5’-ACGCGTCGACCTGCATAAGCAAACTTCGGA-3’ |
| PRE5-BiFC-F1 | 5’-CGCGGATCCATGTCTAACAGAAGATCAAGACAAA-3’ |
| PRE5-BiFC-R1 | 5’-ACGCGTCGACCATGAGTAAGCTTCTAATCACGG-3’ |
| PRE6-BiFC-F1 | 5’-CGCGGATCCATGTCTAGCAGAAGATCATCACG-3’ |
| PRE6-BiFC-R1 | 5’-ACGCGTCGACAACACAGTTAGTTTCGGTCAAA-3’ |
